# Clinical, histopathological, and biomarker characterization of XLMTM and ADCNM: Operational lessons, screening and baseline data of the Unite-CNM study

**DOI:** 10.1177/22143602261471202

**Published:** 2026-08-14

**Authors:** Olga Prikhodko, Tatyana A. Vetter, Sophie Colombo, Chris Freitag, Dominique Nijkamp, Louise Eyler, Leen Thielemans, Jonathan Baets, Mads Stemmerik, John Vissing, Ros Quinlivan, Michela Guglieri, Federica Montagnese, Benedikt Schoser, Frederik Braun, Ulrike Schara, Kris E. Leeuwenberg, Nens van Alfen, Michael W. Lawlor, Belinda S Cowling, Nicol C. Voermans

**Affiliations:** 1 Ionis Pharmaceuticals, Carlsbad, CA, Essen, USA; 2 Diverge Translational Science Laboratory, Milwaukee, WI, USA; 3 Dynacure SA (now Flamingo Therapeutics NV), Louvain, Belgium; 4 Translational Neurosciences, Faculty of Medicine and Health Sciences, University of Antwerp, Antwerp, Belgium; 5 Laboratory of Neuromuscular Pathology, Institute Born-Bunge, University of Antwerp, Antwerp, Belgium; 6 Neuromuscular Reference Centre, Department of Neurology, Antwerp University Hospital, Antwerp, Belgium; 7 Copenhagen Neuromuscular Center, Department of Neurology, Rigshospitalet, University of Copenhagen, Copenhagen, Denmark; 8 Centre for Neuromuscular Diseases, UCL Institute of Neurology, 98546 National Hospital for Neurology and Neurosurgery, London, UK; 9 John Walton Muscular Dystrophy Research Centre, 5994 Newcastle University and Newcastle Hospitals NHS Foundation Trust, Newcastle upon Tyne, UK; 10 Friedrich-Baur-Institut, Department of Neurology, Ludwig Maximilian University (LMU), Munich, Germany; 11 Department of Pediatric Neurology, Centre for Neuromuscular Disorders, Center for Translational Neuro and Behavioral Sciences, 27170 University Duisburg-Essen, Germany; 12 Department of Neurology, Donders Institute for Brain, Cognition and Behavior, 6034 Radboud University Medical Center, Nijmegen, The Netherlands; 13 Department of Neurophysiology and Department of Neurology, Donders Center for Cognitive Neuroimaging, 6034 Radboud University Medical Center, Nijmegen, The Netherlands

**Keywords:** muscle biopsy, freezing artifact, ultrasound, X-linked myotubular myopathy, centronuclear myopathy, clinical trial, image analysis, histopathology analysis, goal attainment scale

## Abstract

**Background:**

X-linked myotubular myopathy (XLMTM) and autosomal dominant centronuclear myopathy (ADCNM) are rare neuromuscular disorders characterized by severe weakness and respiratory impairment and have recently been the focus of therapeutic development. Robust baseline data are essential to understand disease trajectories and enable trial readiness. We present baseline clinical, functional, and biomarker findings from the terminated Unite-CNM trial (NCT04033159), a basket study of the antisense oligonucleotide DYN101, designed to modulate *DNM2* expression in adolescents and adults with XLMTM or ADCNM.

**Methods:**

Screening and baseline evaluations included medical history, patient-reported outcomes, quantitative muscle strength and motor function, respiratory testing, muscle ultrasound and histology, and biomarker analyses (DNM2 protein, creatinine, creatine kinase, cystatin C, myostatin, and microRNAs) in plasma and muscle.

**Results:**

Twenty-six patients were screened (15 *DNM2*, 11 *MTM1*); 14 entered the study. Patient-reported measures highlighted swallowing challenges and variable personal goals. Baseline respiratory impairment was variable in *DNM2* and female *MTM1* patients but consistently reduced in males with *MTM1* (FVC% and FEV1% stable over follow-up to 78 weeks). MFM32 scores were similar across genotypes and sexes, while Myogrip strength was reduced versus age norms yet stable longitudinally. Muscle pathology was comparable across groups. Biomarker analyses showed altered DNM2 protein, reduced myostatin, and specific abnormalities in creatinine, creatine kinase, cystatin C, and microRNAs.

**Conclusion:**

This dataset provides valuable phenotypic, functional, and biomarker characterization of adult XLMTM and ADCNM, emphasizes the importance of baseline data for ultrarare disease trials, and highlights the need for protocol flexibility in response to safety signals and disruptions (e.g. COVID-19).

## Introduction

Centronuclear myopathies (CNMs) including X-linked myotubular myopathy (XLMTM) and autosomal dominant centronuclear myopathy (ADCNM) are causes of severe muscle weakness that have been the focus of several recent therapeutic studies. XLMTM is caused by loss-of-function mutations in *MTM1*, located on the X chromosome, leading to absent or insufficient functional myotubularin, a protein required for normal development, maturation, and maintenance of muscle cells.^1^ XLMTM affects one in 32,000-40,000 newborn males,^2^ but accumulating evidence shows that females can display the disease with various degrees of severity.^3–5^ XLMTM is most notably characterized by profound muscle weakness, resulting in severe respiratory distress at birth in the majority of patients. ADCNM is caused by mutations in *DNM2* that are suspected to lead to a gain of function of Dynamin 2, a large ubiquitous GTPase protein with membrane fission and tubulation activities,^6^ resulting in progressive difficulty with ambulation. Ptosis, ophthalmoparesis, facial weakness, dysphagia, and respiratory insufficiency are commonly reported.^7^ A third main form of CNM, autosomal recessive centronuclear myopathy (ARCNM), is caused by mutations in *BIN1*, encoding the Amphiphysin 2 protein, a membrane remodeling protein.

Preclinical evidence suggests that *MTM1*, *BIN1* and *DNM2* function in the same cellular pathway,^6,8,9^ and that all three forms of CNM display elevated *DNM2* expression and/or activation in muscle.^6^ These advances in the understanding of the pathophysiology of CNM have led to preclinical studies exploring promising approaches to therapy, including antisense oligonucleotides targeted against *DNM2*, and AAV-mediated gene therapy to overexpress *MTM1*. In animal studies, these approaches led to prevention and/or reversal of disease, including improved muscle pathology and strength, greater motor activity and prolonged survival, in various CNM genetic models.^8,10–14^ In parallel, a number of natural history studies were initiated to prepare for future interventional clinical trials, which increased our knowledge of the phenotypic spectrum of different CNM forms.^15–18^

Launched in 2017, the ASPIRO clinical study (NCT03199469) evaluated the investigational AAV8 gene replacement therapy resamirigene bilparvovec (AT132, Astellas Gene Therapies, formerly Audentes Therapeutics), delivering *MTM1* in pediatric patients with XLMTM. Despite being halted in 2021 because of fatal adverse events associated with liver toxicity, the results in the surviving patients showed reduced ventilator dependence and improved motor function,^19^ which correlated with improved muscle histopathological features, including improved organelle localization and myofiber size.^20^

In 2020, Dynacure launched the Unite-CNM trial (NCT04033159) to evaluate the generation 2.5 cEt antisense oligonucleotide DYN101, aimed at reducing *DNM2* expression, in patients with XLMTM and ADCNM. However, the trial was terminated early in 2022 due to tolerability issues.^21^ In this paper, we present the findings from the screening and baseline visits of both the enrolled patients and the screen failures in the Unite-CNM trial. The detailed study protocol provided a systematic assessment of many aspects of these rare myopathies, including clinical outcome measures (muscle grip strength; muscle function (MFM32); eating/swallowing questionnaire (EAT-10); and Goal Attainment Scale (GAS)), muscle ultrasound, histopathological findings of the baseline muscle biopsies, and blood tests. We evaluated the expression of the proteins *DNM2* and Myostatin, as well as several miRNAs linked to muscle function (myomiRs) in muscle and blood and identified several biomarkers of interest. This is the first time that an investigational trial has evaluated both adult ADCNM and XLMTM patients (males and females), the latter presenting a milder form of disease compared to the usually severe phenotype seen in male pediatric XLMTM patients. In addition, the use of core muscle biopsies in this study offers the opportunity to share learnings related to sample procurement and handling that are useful in the planning of future therapeutic studies of skeletal muscle disorders. As a large proportion of samples in this study also encountered muscle biopsy analysis challenges due to freezing artifacts, the analysis performed here also provides an example of how analysis can be performed on suboptimal muscle biopsy specimens.

## Results

### Study subjects

Twenty-six patients were screened for the Unite-CNM study, 15 with *DNM2* mutations and 11 with *MTM1* mutations (Table 1).^21^ Of this cohort, a total of 14 subjects were randomized for inclusion in the interventional study. None of the subjects completed the study, which was terminated by sponsor prior to completion. Twelve subjects were screened but did not fulfill the inclusion criteria or had one or more exclusion criteria, for example liver steatosis due to obesity (Supplementary Table 8). This manuscript focuses on the natural history data from the twenty-six patients screened for the Unite-CNM study. Eight patients (5:3, *DNM2*:MTM1) were previously enrolled in the prospective natural history study (NatHis-CNM; NCT03351270, NCT04977648). An additional 18 patients (10:8, *DNM2*:MTM1) were screened for entry into Unite-CNM, and had 1-3 data points collected during the run-in phase of the study. Of note, at the final timepoint for analysis presented here, patients enrolled in Unite-CNM also underwent a muscle biopsy and blood collection, which were analyzed and are presented in this study.

**Table 1.** Demographics of the subjects screened for the Unite-CNM trial.

Patient #	Gender	Mutation	Age	Ethnicity	Body mass index in kg/m^2^	NHS or run-in	Cohort	Muscle biopsy collected
1	F	*DNM2*	34	White	18.7	NHS	1	Yes
2	M	*DNM2*	51	White	27.9 (OW)	NHS	1	Yes
3	M	*DNM2*	59	White	25.3 (OW)	NHS	1	Yes
4	F	*MTM1*	52	Not Reported	36 (OB)	NHS	1	Yes
5	M	*MTM1*	19	White	20.2	NHS	1	Yes
6	M	*MTM1*	24	White	17.4	NHS	1	Yes
7	M	*DNM2*	36	White	24.5	Run-in	2	Yes
8	F	*MTM1*	53	Not Reported	31.1 (OB)	Run-in	2	Yes
9	M	*DNM2*	41	White	26.6 (OW)	Run-in	2	Yes
10	M	*DNM2*	54	White	23.4	Run-in	2	Yes
11	M	*MTM1*	20	White	19.9	Run-in	2	Yes
12	F	*MTM1*	58	White	26.4 (OW)	Run-in	2	Yes
13	F	*DNM2*	19	White	25.8 (OW)	NHS	2	Yes
14	F	*DNM2*	18	White	22.1	Run-in	2	Yes
15	F	*DNM2*	64	White	21.2	Run-in	Screen failure	No
16	M	*MTM1*	25	Not Reported	24.3	Run-in	Screen failure	No
17	F	*MTM1*	57	Not Reported	Not Reported	Run-in	Screen failure	No
18	M	*DNM2*	26	Not Reported	Not Reported	NHS	Screen failure	No
19	M	*MTM1*	18	White	Not Reported	Run-in	Screen failure	No
20	M	*DNM2*	19	White	28 (OW)	Run-in	Screen failure	No
21	M	*DNM2*	36	White	Not Reported	Run-in	Screen failure	No
22	F	*MTM1*	61	White	Not Reported	Run-in	Screen failure	No
23	M	*MTM1*	58	White	Not Reported	Run-in	Screen failure	No
24	F	*DNM2*	38	White	Not Reported	Run-in	Screen failure	No
25	F	*DNM2*	20	White	14.8 (UW)	Run-in	Screen failure	No
26	M	*DNM2*	53	White	Not Reported	Run-in	Screen failure	No
Summary *DNM2*	9:6; n=15 (M:F)	*DNM2*	36 [18-64]; n=15	14 white, 1 not reported	24.5 [14.8-28]; n=11	Run-in	14 enrolled 12 screen failure	​
Summary *MTM1*	6:5; n=11 (M:F)	*MTM1*	52 [18-61]; n=11	8 white, 3 not reported	24.3 [17.4-36]; n=7	​	​	​

Summary values are expressed as median [range] for Age and Body Mass Index; n = number of patients analyzed; M, male; F, female. Patient numbers remain the same throughout the manuscript. NHS, natural history study; Age, at time of ICF signature. UW: underweight; OW: overweight; OB: obese. Same patient cohort as presented previously. ^21^

### Medical history related to CNM

The medical history of participants is included in Supplementary Table 1. Conditions considered (indirectly) related to XLMTM or ADCNM were neck or low back pain, myalgia, headache, scoliosis, fracture(s), traumatic brain injury after a fall, respiratory weakness, recurrent respiratory infections, pneumothorax, dysphagia/other gastro-intestinal abnormalities, urinary incontinence, corneal scarring, ptosis and dry eyes secondary to CNM.

### Patient-reported outcomes

The Eating Assessment Tool-10 (EAT-10) is a ten-question validated patient-reported questionnaire designed to assess swallowing difficulties, particularly dysphagia. Data from 18 patients identified self-reported problems in all ten questions, for 1-6 out of 18 patients, per question (Supplementary Table 2). The most flagged issues were ‘swallowing pills takes extra effort’ (6/18 patients), and ‘when I swallow food sticks in my throat’ (5/18).

The goal attainment scale (GAS) is a structured method used here to evaluate personal goals from therapeutic intervention in XLMTM and ADCNM individuals. We received data of the defined GAS from 21 subjects, resulting in a total of 105 goals (2 – 11 per subject; mean 5.0; median 5) (Supplementary Table 3). Most goals were in the muscle motor function domain (n=88), followed by the respiratory domain (n=15), and swallowing domain (n=2). The way the goals were described was variable, from very general to very specific and measurable. Examples of very general goals were: ‘more endurance’; ‘climbing stairs’; and ‘walking stability’. Examples of specific and measurable goals were: ‘I want to be stand up from a sitting position on the floor without support of a chair’; ‘I want to be able to walk 60 m without supporting my pelvis with my hand’; and ‘Reduction of exertional dyspnea (SMART: walking up two floors without dyspnea)’. Rating of importance, difficulty and ability at baseline was incompletely performed in four centers (Copenhagen, London, München and Nijmegen).

### Baseline pulmonary function

**Figure 1.**
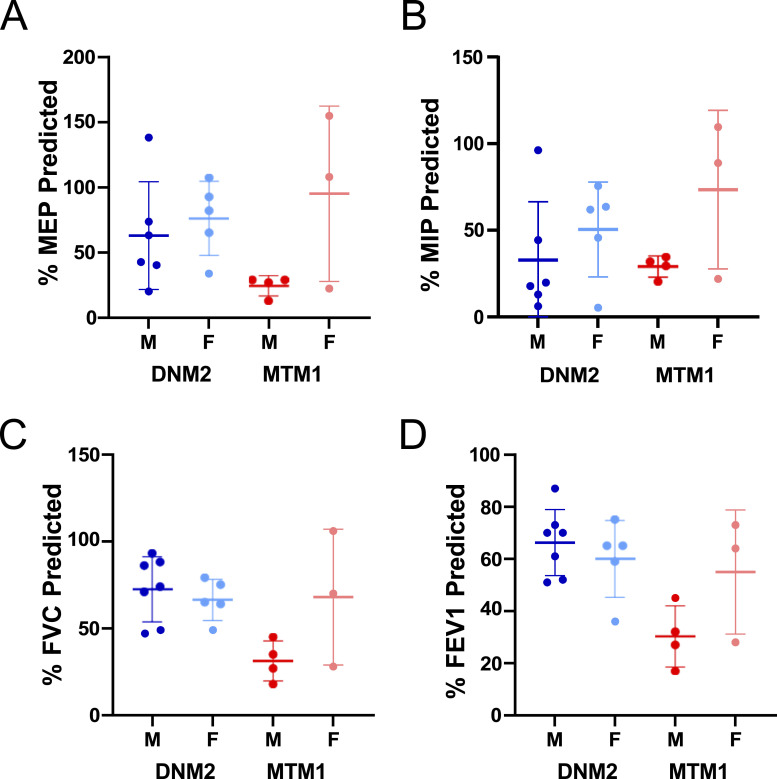
Baseline pulmonary function as percent predicted for age in individuals with DNM2 or MTM1 mutations. (A) Maximum expiratory pressure (MEP, % predicted): DNM2 males (M), 63 ± 42; DNM2 females (F), 76 ± 28; MTM1 M, 24 ± 8; MTM1 F, 95 ± 67. Data are presented as mean ± standard deviation. (B) Maximum inspiratory pressure (MIP, % predicted): DNM2 M, 33 ± 34; DNM2 F, 50 ± 27; MTM1 M, 29 ± 6; MTM1 F, 73 ± 46. (C) Forced vital capacity (FVC, % predicted): DNM2 M, 73 ± 18; DNM2 F, 66 ± 12; MTM1 M, 31 ± 11; MTM1 F, 68 ± 39. (D) Forced expiratory volume in 1 second (FEV1, % predicted): DNM2 M, 66 ± 13; DNM2 F, 60 ± 15; MTM1 M, 30 ± 12; MTM1 F, 55 ± 24.

**Figure 2.**
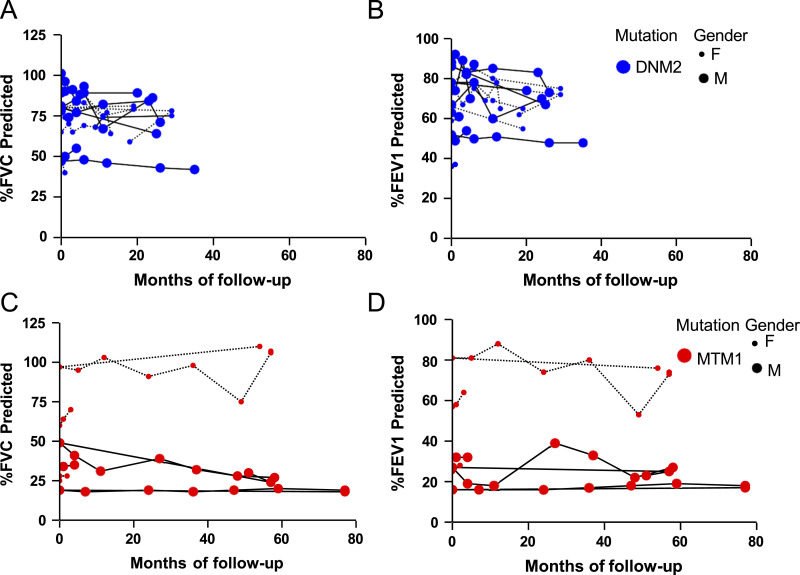
Longitudinal pulmonary function in percentage of predicted value for age of individuals with mutations in DNM2 or MTM1. % Forced vital capacity (FVC) in individuals with DNM2 (A) or MTM1 (C) mutations. % Forced expiratory volume (FEV1) in individuals with DNM2 (B) or MTM1 (D) mutations. DNM2 individuals – blue dots, MTM1 individuals - red dots, females - small dots, dashed line, males – large dots, solid line.

Respiratory function was analyzed, and cross sectional maximum expiratory pressure (MEP%) (Figure 1(a)), maximum inspiratory pressure (MIP%) (Figure 1(b)), forced vital capacity (FVC%) (Figure 1(c)) and forced expiratory volume (FEV1%) (Figure 1(d)) data is shown, represented as a percentage of predicted value by age of individuals. Results were variable in individuals with *DNM2* mutations in both males and females. *MTM1* female individuals, albeit a limited cohort size (n=3), were also highly variable, whereas *MTM1* males showed consistently reduced function across individuals. Forced vital capacity (FVC%) and forced expiratory volume (FEV1%) remained relatively stable overtime, based on prospectively collected longitudinally data for up to 78 weeks in *DNM2* and *MTM1* male and female individuals (Figure 2). Limited longitudinal MIP% and MEP% data was available for comparison (Supplementary Figure 1).

### Muscle strength and motor function of individuals with mutations in DNM2 or MTM1

**Figure 3.**
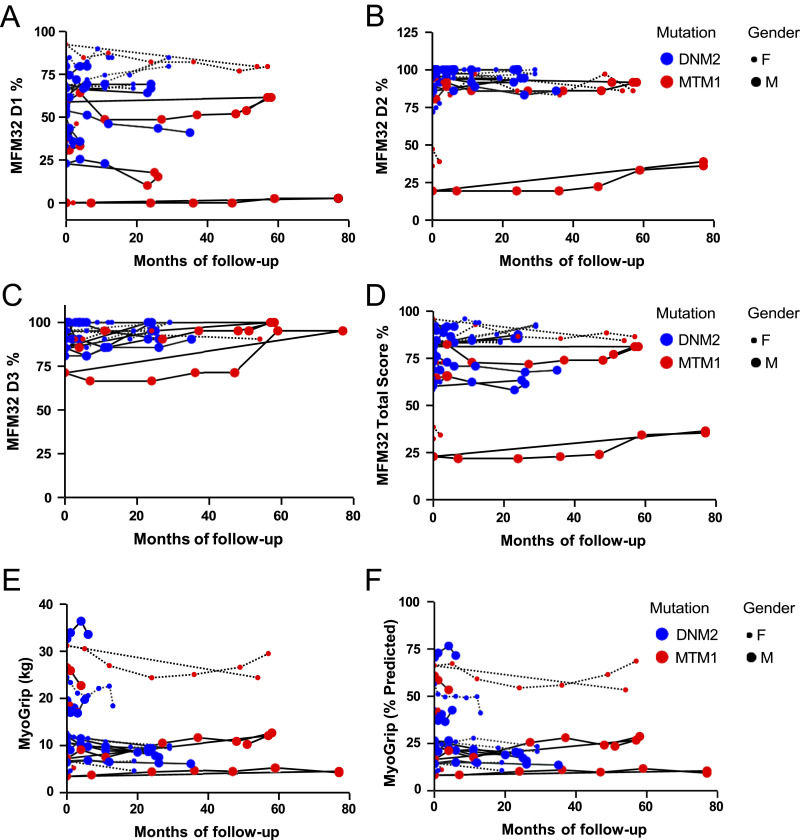
Longitudinal muscle strength and motor function of individuals with mutations in DNM2 or MTM1. (A) %MFM32 D1, (B) %MFM32 D2, (C) %MFM32 D3, (D) %MFM32 Total, (E) MyoGrip (F) MyoGrip expressed as % predicted, across follow-up visits. Screening data is shown, except for Patient 18, for whom the earliest NHS visit is shown instead due to lack of a screening visit.

Motor function measure (MFM32) and Myogrip measurements were performed in individuals at baseline (Supplementary Figure 2), and longitudinally for up to 78 weeks (Figure 3). MFM32, standing and transfers (MFM32 D1), was highly variable across all groups (Supplementary Figure 2A). MFM32, axial and proximal mobility (MFM32 D2), was minimally affected in *DNM2* patients and more variable in *MTM1* individuals (Supplementary Figure 2B). MFM32, distal motor ability (MFM32 D3) was the least affected domain across all groups (Supplementary Figure 2C). The total combined MFM32 score across patients was similar between male and female individuals with *DNM2* or *MTM1* mutations (Supplementary Figure 2D). Myogrip was performed with an electric dynamometer used to measure isometric grip strength in individuals. Myogrip raw values (Supplementary Figure 2E), and percentages of predicted value by age of individuals (Supplementary Figure 2F), highlight no differences between groups, and all groups show significant reduction in Myogrip strength compared to age-normalized expected values. As for pulmonary function, longitudinal analysis of patients for up to 78 weeks showed all domain scores for MFM32 and Myogrip muscle function were relatively stable over time (Figure 3(a) and (d)).

### Muscle ultrasound

In total, 22 muscle ultrasound examinations were performed on 19 subjects, including three subjects who had two ultrasounds performed at different timepoints. The analysis of the images was qualitative in four, semi-quantitative in 11, and both quantitative and semi-quantitative in seven subjects (Supplementary Table 4). Biceps brachii and/or vastus lateralis muscles were measured on both sides in all subjects. Muscle ultrasound images were abnormal in all muscles in every subject. Quantitative analysis resulted in high Z-scores, reported as highly increased echo intensity (EI); semi quantitative analysis resulted in Heckmatt scores of three or four. At the site in Nijmegen, quantitative analysis was performed in more muscles, including the D, TA and GM, as described (Supplementary Table 4).

Muscle ultrasound was subsequently used to guide tissue sampling (needle or open biopsy, at the discretion of the site) from the vastus lateralis or biceps brachii muscles, using a standard operating procedure to ensure image quality (**See annex 1 of** supplementary information).

### Histological evaluation of muscle biopsies

*Visual Assessment of Pathological Findings:* A summary of pathological findings in baseline biopsies is shown in Table 2. Biopsies were attempted on 14 patients, but biopsy sample #6 contained only fat and fibrous tissue and so this sample did not yield useful histology data. There were additional sample quality issues noted on other biopsies, including one sample (#12) that was too small for most evaluations. Several samples showed decreased muscle area due to fatty infiltration, and five biopsies (#1, #2, #7, #10, #14) were noted to have severe or very severe freezing artifacts that affected some analyses. Where freezing artifacts were present, there were usually at least small areas of the biopsy that were of sufficient quality to allow for visual evaluation/estimation of pathological features. However, given these assorted issues, it is worth noting that analysis of seven of the 14 baseline biopsies was substantially impacted by sample quality or handling. As noted below, the biopsy freezing issues offered the opportunity to develop new automated analysis tools that allowed for the measurement of most samples despite the presence of significant freezing artifacts in these samples.

**Table 2.** Histopathological findings in individual muscle biopsies.

Patient	Biopsy tissue	Biopsy type	Est. % Muscle tissue	Fiber size abnormalities	% Internally nucleated fibers	% of fibers with organelle mis-localization	CNM pathology ScoreA	Technical Issues	Interpretation
1	Vastus lateralis	Open	66%	Markedly large and small fibers present	90.51	27% overall (20% punctate, 6% MTM type, 1% radial)	5/5, due to pathology in ∼100% of fibers	Moderate to severe freezing artifact	Centronuclear myopathy
2	Vastus lateralis	Open	90%	Rare small fibers, otherwise appropriate	57.94	30% overall (15% punctate, 15% MTM type)	4/5, due to pathology in 61-80% of fibers	Moderate to severe freezing artifact	Centronuclear myopathy and neuropathic features
3	Vastus lateralis	Open	55%	Markedly large and small fibers present	57.68	15% overall (14% punctate, <15 MTM type, <1% core-like	5/5, due to pathology in ∼100% of fibers	Mild to moderate freezing artifact	Centronuclear myopathy
4	Vastus lateralis	Needle	75%	Markedly large and small fibers present	52.66	12% overall (3% punctate, 8% MTM type, 1% core-like)	3/5, due to pathology in 31-60% of fibers	Very small biopsy area	Chronic myopathic features
5	Vastus lateralis	Needle	60%	Moderately large and small fibers present	76.32	6% overall (6% punctate)	4/5, due to pathology in 61-80% of fibers	Fatty infiltration	Centronuclear myopathy
6	Vastus lateralis	Needle	0%	N/A	N/A	N/A	N/A	No muscle present	No muscle present
7	Vastus lateralis	Needle	50%	Subset of hypertrophic fibers, and then remainder are very small	64.60	33% overall (33% punctate)	5/5 due to pathology in >80% of fibers	Extensive fatty infiltration and severe freezing artifact	Centronuclear myopathy and neuropathic features
8	Vastus lateralis	Needle	90%	Subset of small fibers	75.70	3% overall (1% punctate, 1% subsarcolemmal aggregate, 1% targetoid)	3/5, due to pathology in 31-60% of fibers	Extremely small sample, mild to focally moderate freezing artifact	Chronic myopathic features
9	Bicep brachii	Open	95%	Markedly large and small fibers present	71.84	55% overall (30% punctate, 25% MTM type)	4/5 due to pathology in 60-80% of fibers	None	Centronuclear myopathy
10	Bicep brachii	Open	90%	Rare small and large fibers, mostly normal	66.98	20% overall (17% punctate, 3% MTM type)	4/5, due to pathology in 61-80% of fibers	Very severe freezing artifact	Centronuclear myopathy
11	Vastus lateralis	Needle	95%	Many large and moderately small fibers	78.93	11% overall (11% punctate, <1% targetoid)	4/5 due to pathology in 60-80% of fibers	None	Centronuclear myopathy
12	Vastus lateralis	Needle	ND	Markedly large and small fibers present	74.12	None	5/5 due to pathology in >80% of fibers	Inadequate biopsy size for most analyses. Handling artifacts also present	Centronuclear myopathy and neuropathic features
13	Vastus lateralis	Needle	95%	Markedly large and small fibers present	80.53	33% overall (33% punctate, <1% MTM type)	5/5 due to pathology in >80% of fibers	Moderate freezing artifact	Centronuclear myopathy
14	Vastus lateralis	Needle	95%	Markedly large and small fibers present	73.87	22% overall, (21% punctate, 1% MTM type)	4/5, due to pathology in 61-80% of fibers	Very severe freezing artifact	Centronuclear myopathy

*Note.* Freezing artifact encountered on biopsies #1, #2, #3, #7, #8, #10, #12, #13, and #14 impaired the use of automated assessment tools for the quantification of internally nucleated fibers. For these samples, visual estimates are provided.

**Figure 4.**
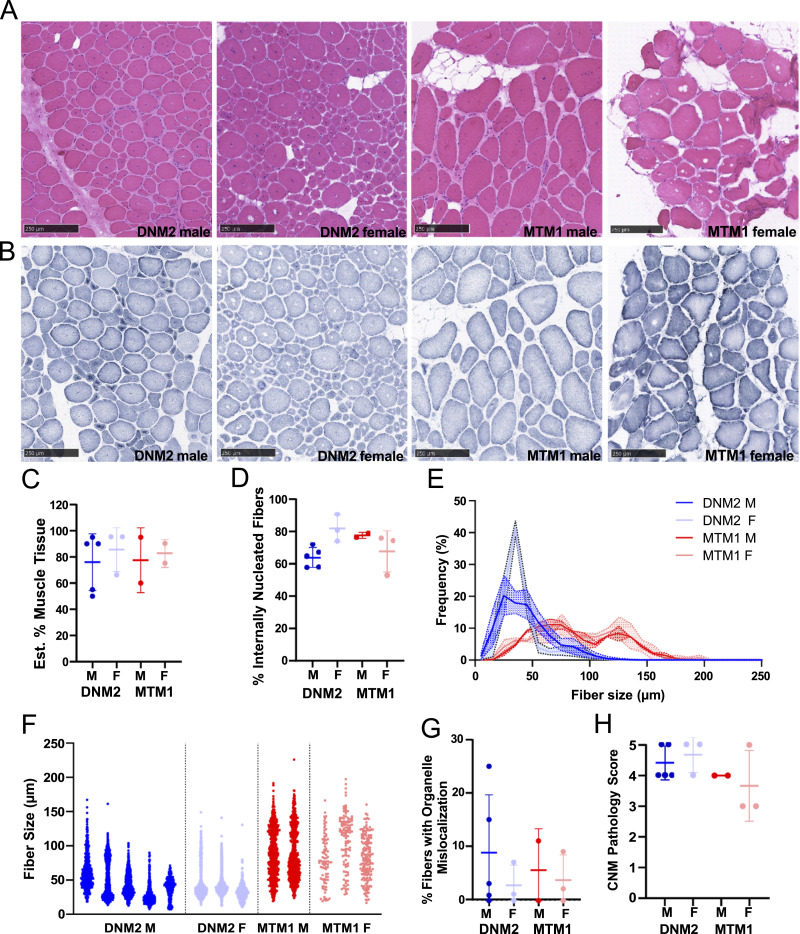
Histological evaluation of muscle biopsies from individuals with DNM2 or MTM1 mutations. (A) Representative hematoxylin and eosin (H&E) staining in muscle sections. (B) Representative NADH staining in muscle sections. (C) Muscle area (% of section): DNM2 M, 76 ± 22 (N=5); DNM2 F, 85 ± 17 (N=3); MTM1 M, 78 ± 25 (N=2); MTM1 F, 82 ± 11 (N=2). (D) Internally nucleated fibers (% of total fibers): DNM2 M, 64 ± 6 (N=5); DNM2 F, 82 ± 8 (N=3); MTM1 M, 78 ± 2 (N=2); MTM1 F, 67 ± 13 (N=2). (E, F) Fiber size distribution (%) across genotypes and sexes. (G) Fibers with non-punctate organelle mislocalization (% of total fibers): DNM2 M, 9 ± 11 (N=5); DNM2 F, 3 ± 4 (N=3); MTM1 M, 5 ± 8 (N=2); MTM1 F, 4 ± 5 (N=2). (H) CNM pathology score: DNM2 M, 4 ± 1 (N=5); DNM2 F, 5 ± 1 (N=3); MTM1 M, 4 ± 0 (N=2); MTM1 F, 4 ± 1 (N=2). Data are presented as mean ± standard deviation. Scale bar = 250 µm.

With respect to pathological findings, 11/13 biopsies containing skeletal muscle tissue showed a moderate to severe degree of pathology that is associated with centronuclear myopathy (including internally located nuclei in 53-91% of myofibers, excessive myofiber size variation, small myofibers, and an absence of myofiber degeneration or active myofiber regeneration (i.e. basophilic fibers on H&E stain; representative images are shown in Figure 4(a)). As shown in Figure 4, the degree and type of pathology related to centronuclear myopathy observed was similar in all biopsies of both genotypes (XLMTM vs. ADCNM) and when comparing males and females. Three of the CNM biopsies (#2, #7, and #12) also displayed nuclear bags, which are most commonly associated with neurogenic atrophy. Two biopsies (#4, #8) did not show findings diagnostic for CNM but rather showed moderate myopathic features including excessive myofiber size variation and a smaller/more focal population of internally nucleated fibers that can be observed in other disorders in addition to CNM. These biopsies were very small, and one had mild to moderate freezing artifacts, so this difference in apparent pathological severity may in part be due to sampling issues or the ability to detect disease-specific findings. All biopsies containing skeletal muscle displayed some level of organelle mislocalization that is consistent with CNM. None of the muscle biopsies displayed evidence of inflammation on H&E or CD3 stains. Key pathological endpoints for each biopsy are shown in Table 2.

### Automated assessment of myofiber size and internal nucleation

Myofiber size and internal nucleation were quantified using automated methods, but automated analysis was significantly impaired in some samples due to the presence of freezing artifacts (Figure 4(a) and (b), Supplementary Figure 3). The degree of freezing artifact particularly affected the quality of the data that could be obtained on the automated quantification of internalized nuclei, but it was possible to develop a specialized semi-automated analysis approach to allow the quantification of at least a portion of each biopsy (Table 2). Freezing artifact was handled by adding a manual annotation step ahead of automated quantification to selectively exclude biopsy regions where artifact was too severe, and to assist in separating myofibers that otherwise may not have been automatically detected as separate. Furthermore, any holes contained entirely within myofibers were filled prior to collecting size measurements and assessing internal nucleation, although nothing could be systematically done to correct for freezing artifact holes around the edges of fibers. When assessing the muscle portion of the image (Figure 4(c)), internalized nuclei were observed (Figure 4(d)). With respect to fiber size, it is often possible to obtain useful fiber size data even when freezing artifacts are present. Individual fibers displayed abnormalities that appear to be related to genotype. While CNM cases due to *DNM2* mutation had a large proportion of very small fibers, enrolled XLMTM patients due to *MTM1* mutations showed a very broad distribution of fiber sizes including very small and very large fibers (Figure 4(e)). This fiber size phenotype may be more characteristic of patients with milder XLMTM that would satisfy the enrollment criteria for this study.

### Manual quantification of organelle mislocalization

Organelle mislocalization was scored manually through the assessment of 200x fields and the assignment of abnormal fibers to different mislocalization classes on NADH-stained slides (Figure 4(b)). Scored percentages of different types of mislocalization are included for each biopsy in Table 2. By far the most common form of mislocalization in the study population was “punctate” mislocalization that corresponds to a small aggregation of mitochondria that is often seen adjacent to an internally placed nucleus (Figure 4(g)). This finding is most likely to be a consequence of the internal nucleation seen in CNM and XLMTM cases rather than a primary problem with organelle localization, and it was also observed in XLMTM biopsies treated with AAV8-MTM1 gene therapy in cases where internal nucleation was still observed following treatment.^20^ As this pattern of mislocalization appears to be nonspecific and related to a different pathological finding that is quantified separately, analysis and data presentation of organelle mislocalization (Figure 4(f)) were focused on “non punctate” organelle mislocalization that related more directly to primary disease pathology. Patterns of non-punctate organelle mislocalization more characteristic of CNM and XLMTM included “XLMTM-type” organelle mislocalization (corresponding to a central aggregate of organelles with a subsarcolemmal “halo” of essentially absent staining, usually in very small fibers), and “radial” organelle mislocalization (corresponding to a radial arrangement of organelles, often radiating from a central area that included a nucleus). There were also a few fibers in some specimens that displayed “core like” mislocalization (corresponding to an area of negative signal in the cytoplasm that likely correlates to an area devoid of mitochondria), “targetoid” mislocalization (corresponding to a decrease in staining intensity in the center of fibers), and subsarcolemmal aggregation of mitochondria. These patterns are more characteristically observed in other disorders (core myopathies, neurogenic changes, and mitochondrial myopathies) when they are especially numerous but can also be observed at low levels in congenital myopathies like CNM as well. Overall, non-punctate organelle mislocalization was highly variable across different genotypes and sexes and there was extensive overlap between datasets (Figure 4(g)).

### CNM pathology score

A “CNM Pathology Score” was applied to each sample as a composite score to reflect the proportion of fibers in the tissue displaying pathological features that are characteristic of CNM. This approach had previously been used in animal models and human studies of XLMTM (and in that context was termed “XLMTM Pathology Score”,^20,22^ and the scored results for each sample are displayed in Table 2. In general, all samples had high CNM pathology scores (scores of 3/5 to 5/5, corresponding to 30-100% of fibers showing pathological findings characteristic of CNM). There was no clear relationship between genotype or sex and CNM pathology score, although the two samples with the lowest overall CNM pathology score were females with *MTM1* mutations (Figure 4(h)).

### Muscle and kidney function tests

As skeletal muscle function is significantly altered (Supplementary Figure 2, Figure 3) and corresponding muscle histopathology was abnormal (Figure 4), we next investigated the following analytes in blood to assess muscle and kidney function: Creatinine, Creatine Kinase, Creatine Kinase-MB, and Cystatin C. We observed that some patients had low Creatinine levels (highlighted in blue), and high Creatine Kinase and Cystatin C levels (highlighted in red) (Supplementary Table 5).

### DNM2 protein expression in muscle biopsies and plasma

**Figure 5.**
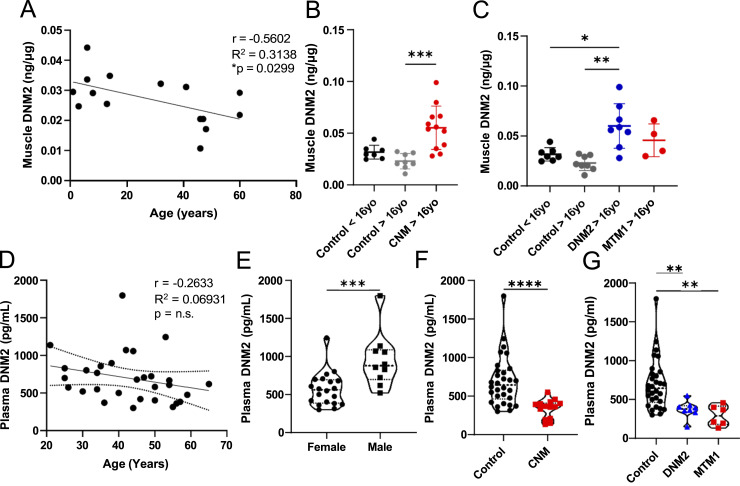
DNM2 protein expression in muscle biopsies and plasma from individuals with DNM2 or MTM1 mutations. (A) DNM2 protein levels in muscle from a cross-sectional control cohort. (B) Muscle DNM2 levels (ng/µg) in control young (0.0316 ± 0.0067, N=7), control adults (0.0229 ± 0.0075, N=8), and adult CNM individuals (0.0663 ± 0.0210, N=12; *p=0.0004 vs control adults, Dunnett’s T3 multiple comparisons test). (C) Same comparison as in (B), with CNM individuals stratified by mutation: DNM2 (0.0601 ± 0.0223 ng/µg, N=8; *p=0.0456 vs control adults; p=0.0085 vs control young, Dunnett’s T3 multiple comparisons test) and MTM1 (0.0457 ± 0.01834 ng/µg, N=4). (D) DNM2 protein levels in plasma from a cross-sectional control cohort. (E) Plasma DNM2 levels in control individuals by sex: females, 570.5 ± 220.9 pg/mL (N=20); males, 950.9 ± 357.6 pg/mL (N=10; *p=0.0008, Mann–Whitney test). (F) Plasma DNM2 levels in controls (697.32887 ± 323.91595 pg/mL, N=30) versus CNM individuals (337.89384 ± 122.28706 pg/mL, N=14; **p<0.0001, Mann–Whitney test). (G) Same comparison as in (F), with CNM individuals stratified by mutation: DNM2 (371.01657 ± 111.85118 pg/mL, N=8; p=0.0088 vs controls, Kruskal–Wallis test) and MTM1 (293.73021 ± 131.41487 pg/mL, N=6; p=0.0031 vs controls, Kruskal–Wallis test).

DNM2 protein and/or activity levels have been shown to be elevated in individuals with *MTM1* or *DNM2* mutations, and in animal models.^23,24^ To better characterize *DNM2* expression, we first analyzed DNM2 protein expression in muscle biopsies from healthy individuals, which showed a significant steady decline in expression with age (Figure 5(a)), with no differences between males and females, and consistent with previously published data.^23^ We then analyzed *DNM2* expression in muscle from skeletal muscle biopsies from individuals with *DNM2* and *MTM1* mutations combined (Figure 5(b), ‘CNM’) or separated (Figure 5(c)). In the combined CNM group, *DNM2* was elevated in patients compared to age-matched controls (∼2.4-fold increase), supporting previous data suggesting *DNM2* was elevated in CNM, and the rationale to reduce *DNM2* as a potential therapeutic target. When analyzed separately, the difference was only significant in the DNM2 CNM group (Figure 5(c)). We next investigated whether *DNM2* could be measured in plasma and serve as a potential biomarker for *DNM2* tissue expression. *DNM2* was detectable in plasma from healthy individuals, with no significant differences observed based on age (Figure 5(d)). Interestingly a significant increase was observed in males compared to females (Figure 5(e)). Importantly when *DNM2* expression was analyzed in plasma from individuals with *DNM2* and *MTM1* mutations combined (Figure 5(f), ‘CNM’), or separated (Figure 5(g)), a significant reduction in *DNM2* was observed, suggesting an inverse relationship between skeletal muscle and plasma levels of *DNM2*. Additional studies would be needed to better understand this relationship, as well as the utility of *DNM2* in plasma as a potential biomarker.

### Investigation of additional biomarkers in blood and muscle

**Figure 6.**
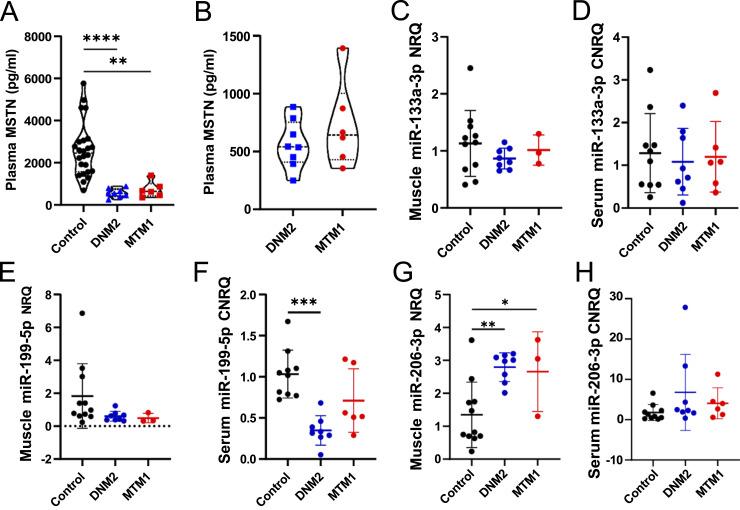
Myostatin and miRNA expression in individuals with DNM2 or MTM1 mutations. (A) Serum myostatin levels in controls (2595.072 ± 1286.247 pg/mL, N=24), DNM2 (562.021 ± 208.096 pg/mL, N=8; **p<0.0001 vs controls, Kruskal–Wallis test) and MTM1 (726.943 ± 372.631 pg/mL, N=6; p=0.0023 vs controls, Kruskal–Wallis test). (B) Same comparison as in (A), with DNM2 and MTM1 groups displayed separately. (C) miR-133a-3p expression in muscle from controls (1.132 ± 0.5781b, N=11), DNM2 (0.8657 ± 0.1816, N=8) and MTM1 (1.014 ± 0.2649, N=3). (D) miR-133a-3p expression in serum from controls (1.285 ± 0.928, N=10), DNM2 (1.082 ± 0.7787, N=8) and MTM1 (1.2 ± 0.8241, N=6). (E) miR-199-5p expression in muscle from controls (1.831 ± 1.963), DNM2 (0.6047 ± 0.3032) and MTM1 (0.4897 ± 0.2858, N=3). (F) miR-199-5p expression in serum from controls (1.033 ± 0.2914), DNM2 (0.3485 ± 0.194, *p=0.0001 vs controls, one-way ANOVA with Dunnett’s multiple comparisons test) and MTM1 (0.711 ± 0.3859, N=6). (G) miR-206-3p expression in muscle from controls (1.347 ± 0.9937, N=11), DNM2 (2.792 ± 0.4380, N=8; p=0.0038 vs controls, one-way ANOVA with Holm–Šidák multiple comparisons test) and MTM1 (2.659 ± 1.211, N=3; *p=0.0307 vs controls, one-way ANOVA with Holm–Šidák multiple comparisons test). (H) miR-206-3p expression in serum from controls (1.789 ± 2.002, N=11), DNM2 (6.783 ± 9.428, N=8) and MTM1 (4.061 ± 3.844, N=6). Data are expressed as mean NRQ (normalized relative quantification) or CNRQ (corrected normalized relative quantification) ± standard deviation.

We first focused on myostatin, a circulating biomarker that is essential for muscle growth.^25^ We previously showed that myostatin levels are reduced in mice and in plasma of CNM patients from the NatHis study and increased in response to therapy in mice.^26^ Here, we confirm that myostatin levels are reduced in plasma of both *MTM1* and *DNM2* patients (Figure 6(a) and (b)) and thus could potentially be used as a disease biomarker for individuals with mutations in *DNM2* or *MTM1*.

Finally, we investigated microRNA expression from skeletal muscle and serum (Supplementary Table 6). The 3′UTR region of the *DNM2* gene contains a seed site for the myomiR: miR-133a. Despite the altered *DNM2* expression in skeletal muscle biopsies and plasma, no differences were observed in adult individuals with *DNM2* or *MTM1* mutations compared to controls (Figure 6(c) and (d)). A second microRNA: miR-199 was investigated, which is encoded in the opposite strand of DNM genes.^27^ A trend for miR-199 reduction was observed in muscle and serum, with a significant reduction observed in individuals with *DNM2* mutations (Figure 6(e) and (f)). Finally, miR-206 is a myomiR acting downstream of myostatin pathway and is inhibited by myostatin activation to regulate muscle growth.^28,29^ miR-206 was significantly elevated in skeletal muscle from individuals with *DNM2* or *MTM1* mutations (Figure 6(g)), however no difference was observed in serum (Figure 6(h)), thus limiting its utility as a circulating biomarker. Additional miRNA biomarkers analyzed (miR-1, miR-22, miR-95, miR-193, miR-486) can be found in supplementary information (Supplementary Figure 4, Supplementary Table 6). The correlations of biomarkers between controls and individuals with CNM and MFM32 score are summarized in Supplementary Table 7, with muscle miR-1-3p being the only significant correlation (p=0.0232).

## Discussion

This report describes the results of the first phases of the Unite-CNM trial - a basket trial of DYN101, an ASO that was designed to modulate *DNM2* expression, in adolescents and adults with XLMTM and ADCNM. Here we present the clinical findings of the screening and baseline visits of study participants and screen failures. The data on clinical outcome measures, muscle ultrasound, muscle histopathology and various biomarkers in blood and muscle provide a detailed description of the phenotype of adult patients with XLMTM and *DNM2*-CNM. Additionally, operational elements of muscle biopsy collection and analysis (use of core muscle biopsies, handling of freezing artifacts) offer important learnings for planning and analysing muscle samples in future clinical trials.

Twenty-six patients were recruited (15 with *DNM2* mutations, 11 with *MTM1* mutations) of which 14 were randomized (screen failure rate 46%). The main reason for screen failure was not meeting enrolment criteria (7/12 subjects; Supplementary Table 7). These criteria excluded patients with significant liver disease due to emerging liver findings in other clinical studies,^21^ and skewed enrollment towards milder phenotypes, particularly in XLMTM; thus, the more severely affected, typically pediatric, patients were not eligible. This greatly restricted the eligible population in an ultrarare indication, and is a significant consideration for future CNM clinical trials. The XLMTM cohort was comprised of six males and five females, with females being mild-to-moderately affected, in line with observations in recent studies on females with XLMTM.^30,31^ This underscores the importance of including females with this X-linked neuromuscular condition in clinical trials, mirroring emerging recognition of affected women with dystrophinopathies.^32,33^

Longitudinal assessment for up to 78 weeks showed that respiratory function and muscle function (all MFM32 domains and Myogrip) were relatively stable over time (Figures 2 and 3). This likely reflects that the cohort included only adults and did not capture the most severely affected children, who do not survive to adulthood. The findings are in line with the international prospective, longitudinal natural history data in XLMTM, in which a 2.3 point decline (2.4%) in MFM32 score over one year was observed in participants older than 6 years.^17^ Interestingly, despite stable functional scores over time, infants in the INCEPTUS run-in study, representing the more severe end of the XLMTM spectrum, had high ventilator dependence, early tracheostomy and substantial mortality.^18^ For ADCNM, the results of the multicentric natural history study (n=15) also showed largely stable muscle strength, function and pulmonary capacity over 36 months, with age-dependent strength trajectories, but no change in ambulation status.^34^

This study provided an important insight into muscle histopathology in adults with XLMTM and ADCNM. Muscle biopsies from 11 of 14 patients showed moderate to severe CNM pathology, including internalized nuclei, fiber size variation, and small myofibers without degeneration or regeneration. Three biopsies exhibited nuclear bags, and three others showed only moderate myopathic changes, likely influenced by sample quality. All samples displayed organelle mislocalization, with no inflammation detected. The combination of myopathic and neuropathic features here is noteworthy, as young CNM children typically show a purely myopathic pattern. This may reflect the older age of patients in this study, allowing the progression of muscle pathology to also incorporate neuropathic disease or comorbidities in these patients. The degree of pathological abnormality was similar when comparing XLMTM to ADCNM, and between males and females, but myofiber size distribution differed; ADCNM patients had high proportions of very small fibers, whereas XLMTM patients showed a broader range including larger fibers. This finding is particularly unusual because many cases of XLMTM contain extremely small fibers, even smaller than what is observed for the *DNM2* patients here. In the authors’ experience, myofiber smallness in CNM is at least partially related to the clinical severity of the patient,^35^ and so the unusually large fiber size encountered in the XLMTM patients in UNITE-CNM may reflect the milder disease of this adult cohort than typical male, pediatric XLMTM patients.

The biomarker data generated in UNITE-CNM complements and extends prior work in CNM. We confirm a ∼2.6-fold elevation of DNM2 protein in skeletal muscle from adult individuals with ADCNM compared to age-matched controls, consistent with earlier reports in patients and animal models^23,24^ and reinforcing the mechanistic rationale for DNM2-lowering approaches. In contrast, DNM2 levels were significantly reduced in plasma despite being increased in muscle, suggesting an inverse relationship between tissue and circulating compartments that complicates the use of plasma DNM2 as a simple surrogate of skeletal muscle target engagement and warrants further longitudinal and mechanistic investigation. At the level of post-transcriptional regulation, we did not observe differences in miR-133a between CNM and controls in either muscle or serum, arguing against a major role for this myomiR as a biomarker in adults, whereas serum miR-199-5p was significantly reduced in individuals with DNM2 mutations, in line with the proposed relationship between miR-199 and dynamin^27^. Finally, miR-206 was markedly increased in skeletal muscle but unchanged in serum, mirroring preclinical data linking myostatin pathway suppression and miR-206 induction^26,28,29^ and indicating that muscle, rather than blood, may be the more informative compartment for miRNA-based biomarkers in CNM. Importantly, these observations are derived from an adult cohort enriched for comparatively milder phenotypes and may therefore not fully represent biomarker behavior across the more severe, early-onset, or classical pediatric XLMTM spectrum. Together, these findings extend prior biomarker studies in pediatric XLMTM and preclinical models^23,24,26^ into an adult CNM population and provide an initial framework for selecting and prioritizing DNM2- and miRNA-based biomarkers in future interventional trials.

The UNITE-CNM study also yielded several operational insights and learnings relevant to trial design in rare neuromuscular disorders. Use of the Goal Attainment Scale (GAS) showed what patients consider meaningful change, with most goals related to motor function, and some reflecting modest strength gains expected to yield strong functional improvement. However, the protocol instructions led to limited definition of specific and measurable goals, echoing a recent review that highlighted the heterogeneous application of GAS across trials and the need for standardization.^36^ Future CNM studies using GAS should include clearer guidance, structured training and, ideally certification of assessors, similar to established clinical outcome scales such as the MFM.

Several technical challenges with muscle biopsy assessment highlight the need for careful site training and quality control. UNITE-CNM allowed open or needle biopsies; nine of 14 baseline samples were needle biopsies, but one (Patient 6) did not contain muscle tissue and another (Patient 12) contained too little muscle for full evaluation. While needle or core muscle biopsies are commonly used in the diagnostic setting, issues here highlight the challenges of using needle biopsies in disorders where muscle mass may be very low. Muscle ultrasound was used in UNITE CNM to select the appropriate biopsy site, but did not reliably prevent collection of insufficient specimens. Nevertheless, ultrasound or MRI remain valuable tools to assess muscle quality, and here showed structural abnormalities even in muscles that still had normal muscle strength or function. Freezing artifacts represented a second major technical issue, with severe or very severe freezing artifacts in 5/13 muscle samples, and milder freezing artifacts in several others. This affected quantitative endpoints including fiber size, internal nucleation, and organelle mislocalization, and required adaptations of the automated image-analysis pipeline together with expert visual review. This was also a concern reported in the ASPIRO trial, where 5/28 biopsies needed to be thawed and refrozen, as the significant freezing artifacts observed would complicate reliable histopathologic and quantitative analysis.^20^ A detailed description of freezing procedures, types of artifact observed, and mitigation strategies is provided in the Supplementary Methods (Muscle biopsy handling and freezing artifact; see also Supplementary Figure 3).

This study has several strengths. The PIs and patient organizations actively encouraged publication of baseline results despite early trial termination, in alignment with the sponsor. Doing so helps to counteract the publication bias in rare diseases, where selective reporting of positive results can distort the scientific literature and hinder evidence-based medicine.^37^ Patient organizations can play a crucial role in preventing publication bias by advocating for transparency, ensuring that both positive and negative clinical trial results are published, and helping to hold researchers and sponsors accountable.^38^ Furthermore, the trial included a relatively large adult CNM cohort spanning from mild to more severely affected, and employed a comprehensive set of clinical, functional, imaging, histopathological and biomarker assessments. The protocol was iteratively revised based on insights from ASPIRO liver safety findings, and in response to to COVID19 constraints. The findings from the ASPIRO trial led to extension of the hepatic screening before inclusion: an ultrasound of the liver was performed before first dosing, and a fibroscan was added. The COVID19 pandemic led to different regulations in different countries throughout the trials, following the subsequent lockdowns due to which inclusion of patients had to be postponed. Furthermore, patients who tested positively for COVID19 also had to postpone their study visits. This illustrates the responsive approach to trial conduct in ultrarare disorders. Follow-up of up to 78 weeks further underscores the value of long-term, systematically collected data, whether through natural history studies or integration of long-term follow-up into routine clinical care.

To conclude, we report the results of the first phases of the Unite-CNM trial, a basket trial of DYN101 for adolescents and adults with XLMTM and ADCNM. The results provide important insights into adult phenotypes of both forms of CNM and highlight key operational considerations for future trials, including endpoint selection, muscle biopsy practice, and adaptive trial design. Publishing these results, despite early termination, maximizes the scientific value of the trial and honors the substantial efforts made by participants and their families.

## Methods

### Unite-CNM study design

This was a Phase 1/2 trial on the safety, tolerability, pharmacokinetics, pharmacodynamics and exploratory efficacy of DYN101 in patients ≥ 16 years of age with centronuclear myopathies caused by mutations in *DNM2* or *MTM1* (https://www.clinicaltrialsregister.eu/ctr-search/trial/2018-004089-33/DK; https://clinicaltrials.gov/ct2/show/study/NCT04033159?term=dynacure&draw=2&rank=2). The investigational medicinal product (IMP) DYN101 is a generation 2.5 cEt gapmer antisense oligonucleotide targeting *DNM2* for degradation. The study was performed in a total of eight sites in six EU countries (Netherlands, Belgium, Germany, France, Denmark, UK). The first subject was enrolled on 04 March 2020. The last subject’s visit was on 08 July 2022. The study was stopped prior to completion due to tolerability issues.

### Clinical assessments

The following clinical assessments were performed: (1) Goal Attainment Scale (GAS, see supplementary information for details); (2) Respiratory function (MEP, MIP, FVC, FEV1); (3) Muscle function (MFM32); (4) Myogrip dynamometer; (5) Eating/swallowing questionnaire (EAT-10).

### Muscle histopathology

Muscle biopsies were obtained from the vastus lateralis following ultrasound assessment of muscle depth and suitability, using either open or needle biopsy per site standard practice. If the vastus lateralis was unsuitable, biopsies were collected from the biceps brachii. Tissue samples were frozen in isopentane cooled in liquid nitrogen according to standard procedures,^39^ shipped to Eurofins Central Laboratory, and subsequently transferred to Diverge Translational Science Laboratory for processing.

*Tissue staining and evaluation:* Upon receipt of muscle tissue for histology assessment, internal blinding identifiers were assigned. For pathological assessment, all samples were cut at 8-µm thickness and stained with H&E, NADH-TR, or immunohistochemically stained for CD3 (Leica PA30553, diluted 1:1) using standard techniques.^40^ Whole slide scans were obtained at the Children’s Hospital of Wisconsin Research Institute’s (CRI) Imaging Core Facility using a Nanozoomer HT2 scanner with NDPI software.

*Tissue analysis:* Tissue histopathology was visually assessed by a Board-Certified Anatomic- and Neuro-Pathologist (MWL) and a broad range of pathologies was scored according to a modified Common Data Elements approach to muscle biopsy reporting.^41^ A CNM pathology score was calculated, as previously described.^20^ Myofiber size and internal nucleation were assessed from slide scans of H&E-stained sections captured in CRI Imaging Core Facility at the Medical College of Wisconsin. Myofiber size and internal nucleation were quantified in H&E images in a semi-automated fashion using NIS-Elements software (v 6.10) with the General Analysis 3 (GA3) software module. Quantification of organelle mislocalization was performed on NADH-TR-stained slides, as described previously.^20^ See supplementary information for more details.

### Laboratory tests in blood

Measurements of Creatinine, Creatine Kinase, Creatine Kinase-MB, and Cystatin C were part of the laboratory safety assessments, using standard lab practices.

### Western Blot

Samples were lysed in Tissue Protein Extraction Reagent (T-PER) (#78510 – Thermo Scientific) buffer with Mix Halt Protease Inhibitor Cocktail (1/100) and Mix Halt Phosphatase Inhibitor Cocktail (1/100) for one hour at 4°C with agitation. Samples were centrifuged at 10,000 rpm for five minutes at 4°C and supernatants were collected. Samples were diluted at 1 ng/µL in Total T-PER buffer, NuPAGE LDS sample buffer (#NP0007 – Invitrogen) with 0.5M DTT and denatured at 70°C for 10 minutes. 12.5 ng of protein, as well as a *DNM2* recombinant protein standard curve (0.5 ng, 0.25 ng, 0.1 ng, 0.06 ng, 0.03 ng, 0 ng) were separated on 4-15% precast polyacrylamide gels. Membranes were blocked for 5 minutes at room temperature (RT) in EveryBlot Blocking Buffer (#12010020 – Biorad), then incubated for one hour at RT with antibodies against *DNM2* (1/1000, #PA5-19800 – Thermo Scientific), or GAPDH (1/1000, #MA515738 – Invitrogen). Following 3 washes with TBST, membranes were incubated with secondary antibodies (1/1000 for GAPDH, #32430 – Invitrogen and 1/1000 for *DNM2*, #32460 – Invitrogen) for one hour at RT. Signal was detected using a Fusion FX imager (Vilber) after an incubation with ECL Immobilon (#WBKLS0100 – Millipore).

### Plasma ELISA sample analysis

Plasma ELISA sample analysis was performed by PBL Assay Science (New Jersey, USA) using R&D Systems, Inc. Quantikine® ELISA GDF-8/Myostatin kit (R&D Systems, Cat # DGDF80) and LifeSpan Biosciences, Inc. Human *DNM2*/Dynamin-2 ELISA kit (LSBio, Cat # LS-F11337). Assay validation is described in the supplementary information. For sample analysis, a total of 45 human plasma study samples from Unite-CNM were tested with both Myostatin and *DNM2* kits. Samples were diluted (myostatin 1:4; DNM2 1:5). The calibration curves were prepared as per the manufacturer’s recommendation and assayed in duplicate on each plate. All samples were processed in triplicate. After performing ELISAs according to the kit instructions, O.D. 450 nm readings were performed using a Spectramax ABS plate reader and utilizing SoftMax Pro Software (Version 7.1) for analysis. Assay standards of known concentration were plotted against their O.D. values, and a 4-PL log fit analysis was applied to create a standard curve for each respective assay. Sample values were backfitted against the standard curve to determine their concentrations, which were then multiplied by their respective dilution factors (DF) to arrive at a final concentration of analyte per sample (pg/ml). The LLOQ (Lower Limit of Quantitation) was determined as the lowest point at which the interpolated data of the standard curve are within 25% (±) of the expected recovery (% Nominal), CV is ≤ 25%, and the signal to noise (S/N) ratio is >1.5. The ELISA validation parameters are included in Supplementary Table 9.

### Serum and skeletal muscle miRNA qPCR

miRNA analysis was performed at BioXpedia. Extraction of total RNA including miRNA was done manually in a centrifuge using the miRNEasy Tissue/Cells Advanced Kit (Qiagen, cat. no. 217684). The RNA received a DNase-treatment prior to elution. The RNA was eluted in 50 µL of RNase-free water (Qiagen). Quantification of the RNA was performed using two different methods; a) the Agilent Bioanalyzer instrument using the Agilent Small RNA kit (to quantify the amount of the miRNA fraction in the samples); and b) Agilent Tapestation using the Agilent RNA screentapes (to quantify the amount of total RNA). The RNA was normalized using the results from the Small RNA kit prior to qPCR. The samples were normalized to 80 pg/uL (based on the miRNA concentration). The cDNA was synthesized and miRNA qPCR expression levels determined following the miRCURY® LNA® miRNA SYBR® Green PCR Handbook (October 2019). Target gene expression was normalized via the geNorm method,^42^ with hsa-miR-361-5p, hsa-miR-425-5p, and hsa-miR-185-5p used as reference genes for serum, and hsa-miR-361-5p, hsa-191-5p and hsa-103a-3p used as reference genes for muscle.

### Statistical analysis

For the *DNM2* Western Blot, *DNM2* and Myostatin ELISA and miRNA qPCR assays, statistical analysis was performed using GraphPad Prism v9.4. The tests performed are indicated in the figure legends.

### Ethics statement

The studies were conducted in accordance with International Conference on Harmonisation Good Clinical Practice guidelines and the Declaration of Helsinki. Study protocols were approved by the regulatory agencies in each country, and subsequently institutional permission was granted by institutional review board/independent ethics committees (IRB/IECs). Signed informed consent forms (ICF) were obtained from participants or legal guardians.

## Supplemental material

Supplemental material - Clinical, histopathological, and biomarker characterization of XLMTM and ADCNM: Operational lessons, screening and baseline data of the Unite-CNM study Supplemental material for Clinical, histopathological, and biomarker characterization of XLMTM and ADCNM: Operational lessons, screening and baseline data of the Unite-CNM study by Olga Prikhodko, Tatyana A. Vetter, Sophie Colombo, Chris Freitag, Dominique Nijkamp, Louise Eyler, Leen Thielemans, Jonathan Baets, Mads Stemmerik, John Vissing, Ros Quinlivan, Michela Guglieri, Federica Montagnese, Benedikt Schoser, Frederik Braun, Ulrike Schara, Kris E. Leeuwenberg, Nens van Alfen, Michael W. Lawlor, Belinda S Cowling, Nicol C. Voermans in Journal of Neuromuscular Diseases
