# Supplementary material for: Clinical, histopathological, and biomarker characterization of XLMTM and ADCNM: Operational lessons, screening and baseline data of the Unite-CNM study
Source: J Neuromuscul Dis. 2026 Aug 14:22143602261471202. Online ahead of print. doi: 10.1177/22143602261471202 (PMC13476381; doi:10.1177/22143602261471202)

Supplementary information

Supplementary methods

Clinical assessments

Goal Attainment Scale (GAS) is a method consisting of the development of a personalized written follow-up guide between the patient and the clinician used to monitor therapy effect [1]. The written instructions included to classify the goals within three domains: muscle motor function, respiratory function, or swallowing. Furthermore, patients were asked to rate the importance (0 – 3; 0 = least important and 3 = most important) and difficulty (1 – 3; 0 = most easy and 3 = most difficult) and indicated their ability at each goal at baseline (some; none). The protocol proposed to assess the effect of the therapy by assessing the subsequent responses on the GAS (as expected; little better and much better) and subsequent comparison of the proportion of GAS Questionnaire responders (responders defined as responding "YES – MUCH BETTER," "YES – A LITTLE BETTER") using the PK Set by dose (cohort), for single or multiple administration, and by mutated gene (subcohort) at each post-baseline visit. No data were received of five patients in Paris (two participants and three screen failures).

Muscle ultrasound: Muscle ultrasound imaging of both the vastus lateralis (VL), and the biceps brachii (BB) if the VL was found inadequate, was performed at screening to determine which muscle should be biopsied. This imaging should not be older than 6 months at baseline. Three subjects had a second muscle ultrasound investigation during rescreening, as their last ultrasound was dated at >6 months before the biopsy was performed. Visual qualitative (normal vs abnormal), semi-quantitative (Heckmatt scale) and quantitative muscle (Z-score) ultrasound tools were allowed to be used to assess the biopsy site [2]. A standard operating procedure was developed to assure image quality [see **Annex 1**], and the participating centers were trained by an expert operator (NvA Radboudumc). After determining the best

biopsy site, images were stored for offline analysis. No data were received of five patients in Paris (two participants and three screen failures) and two patients in NewCastle (both screen failures).

Pathological Evaluation:. Areas of specific focus in reporting included indicators of myofiber degeneration/regeneration (evaluated on H&E stain), abnormalities of myofiber size (evaluated on H&E stain), abnormalities of organelle localization (most easily recognized on NADH stain), and inflammation (evaluated on H&E and CD3 stains). For scoring of the CD3 stains, a grading scale was applied that included the following scores: 0 = Normal levels of positivity, 1 = Mildly increased numbers of CD3 positive cells, 2 = Moderately increased numbers of CD3 positive cells, and 3 = Severely increased numbers of positive cells. Narrative portions of the report also addressed tissue adequacy/quality issues and whether artifacts or tissue quality may render automated analyses unreliable for a given sample. Visual estimates of the percentage of internally nucleated fibers were made for each sample to allow the estimation of this endpoint in situations where automated quantification was thought to be unreliable. All reporting of pathological findings was performed in a blinded fashion, and finalized reports were uploaded to the Sponsor before unblinding.

Measurement of myofiber size and internally nucleated fibers: Each H&E image was initially reviewed for suitability for automated myofiber segmentation, and regions with unacceptably severe freezing artifact, tissue folding, or longitudinal myofiber orientations were manually annotated for exclusion in NIS-Elements. In instances where a minor proportion of the biopsy section was suitable for analysis, the subregion was cropped from the larger image. Following initial artifact exclusion, minor manual binary annotations were added to separate myofibers in regions where automated segmentation was expected to struggle (e.g., a short thin line at a junction where two fibers appear visually indistinct). The annotated images were then processed through an automated analysis algorithm to segment discrete

myofibers, measure fiber size, identify nuclei, and mark and count internally-nucleated fibers. Two different algorithm versions performed myofiber segmentation using one of two analysis approaches, where the best approach depended on the tissue characteristics and challenges: either a combination of conventional thresholding and tight borders detection, or the automated "IHC Classification" tool available in NIS-Elements. In both algorithms, the manual annotation binary layer was then subtracted from automatically segmented myofibers to exclude or separate certain fibers as needed, and myofiber measurements and detection of nuclei followed in an identical fashion.

Quantification of organelle mislocalisation: We have previously assessed organelle mislocalization in XLMTM patients before and after gene therapy, and patterns of mislocalization included XLMTM-type mislocalization (central aggregations of organelles with a subsarcolemmal area of clearing), punctate mislocalization (a small internal aggregation that appears to be a small cluster of mitochondria around a subset of internally located nuclei), radial mislocalization (a radial arrangement of mitochondria that has been described in association with some forms of centronuclear myopathy), and core-like mislocalization (a core-like area devoid of NADH-TR signal) [3-9]. This abnormality is poorly suited to automated quantification methods, so organelle mislocalisation was manually quantified [4, 5]. Briefly, biopsied tissue with NADH-TR stain was photographed using a slide scan image on 200x magnification. The field selected for photography was judged to be an area with the highest degree of organelle mislocalization in each specimen. The image was quantified manually using the Cell Counter tool in ImageJ software, with respect to the number of fibers with appropriate organelle localisation or inappropriate organelle localisation.

CNM pathology score: An pathology scoring system (called “XLMTM Pathology Score” in prior studies focused on XLMTM) had been developed for studies of XLMTM canine and human muscle biopsies to allow an integration of different characteristic pathological features into a single score[3]. Briefly, the score is based on an estimate of the overall proportion of fibers in the biopsy displaying some key elements of characteristic XLMTM pathology (myofiber smallness, organelle mislocalization, or internal nucleation). While it was classified as an “XLMTM Pathology Score” in prior work, this scoring system would be more appropriate to name as a “CNM Pathology Score” in the current study. Grading was assigned as follows: Grade 0, no CNM pathology (normal muscle or muscle with scattered atrophic fibers); Grade 1, very mild CNM pathology (<10% of fibers with CNM pathology); Grade 2, mild CNM pathology (11–30% of fibers with CNM pathology); Grade 3, moderate CNM pathology (31–60% of fibers with CNM pathology); Grade 4, moderate/severe CNM pathology (61–80% of fibers with CNM pathology); and Grade 5, severe/very severe CNM pathology ($\geq$81% of fibers with CNM pathology).

Muscle biopsy handling and freezing artifact: Freezing artifact is a specific challenge in the pathological assessment of skeletal muscle tissue because 1) skeletal myofibers are large in comparison to most cell types, with a high water content, 2) most diagnostically relevant histological findings (and many stains required for the recognition of skeletal muscle pathology) require the use of frozen tissue rather than fixed tissue. Freezing artifact arises in skeletal muscle when the speed of myofiber freezing is too slow to prevent ice crystal formation, and the optimal procedure to avoid freezing artifact involves the use of freezing isopentane that has been cooled in a bath of liquid nitrogen. Other freezing strategies (freezing in liquid nitrogen, using dry ice, or in a freezer) result in slower freezing and the formation of large ice crystals at the center of myofibers (**Supplementary figure 2**). These crystals can cause architectural distortion in the muscle or the sarcoplasm and affect the recognition or measurement of pathological findings. In this study, endpoints that were affected by freezing artifact included assessment of fiber size,

internal nucleation, and organelle mislocalization. It was possible to still evaluate some of these endpoints based on the authors' experience and the use of specialized automated analysis approaches, but the optimal situation would involve appropriate site training, screening for freezing quality at regular intervals during the study, and periodic retraining of site staff. It should also be noted that it is possible to recover a large degree of the structural consequences of improper freezing using a thawing/refreezing approach, but that is not always possible when biopsies are small or where thawing/refreezing would affect some of the intended endpoints for measurement.

Supplementary tables

Supplementary table 1. Medical history of patients.

Medical history related to CNM	XLMTM		ADCNM		Total
	**Male**	**Female**	**Male**	**Female**	
Neck or low back pain	1	1			2
Myalgia			1		1
Headache				1	1
Scoliosis	1	1			2
Fracture(s)	1				1
Traumatic brain injury after a fall				1	1
Respiratory weakness	2	1		1	4
Recurrent respiratory infections	1				1
Pneumothorax		1			1
Dysphagia / other gastrointestinal abnormalities	2			1	3
Urinary incontinence				1	1
Corneal scarring	1				1
Ptosis	1	1	1		3
Dry eyes secondary to CNM	1				1
Neuropathic pain			1		1
Paresis / hypomimia			1		1
Diplopia		1			1
Knee pain		1			1

Supplementary Table 1: **Medical history of patients**. Medical history of patients was reported by their physician, each number represents the number of patients for which that symptom was recorded.

Supplementary table 2. EAT-10 Questionnaire.

**Number of patients answering:**	**0 = No problem at all**	**1**	**2**	**3**	**4**
**1. My swallowing problem has caused me to lose weight.**	17	1			
**2. My swallowing problem interferes with my ability to go out for meals.**	17		1		
**3. Swallowing liquids takes extra effort.**	17	1			
**4. Swallowing solids takes extra effort.**	15	2		1	
**5. Swallowing pills takes extra effort.**	12	4	2		
**6. Swallowing is painful.**	17	1			
**7. The pleasure of eating is affected by my swallowing.**	17			1	
**8. When I swallow food sticks in my throat.**	13	4	1		
**9. I cough when I eat.**	14	4			
**10. Swallowing is stressful.**	16	2			

Supplementary table 2. Results of EAT-10 questionnaire. Table boxes contain the number of patients who responded with either 0 = no problem at all; 1 = mild problem; 2=mild to moderate; 3 = moderate; and 4 = severe problem, to the EAT-10 questions listed in row 1.

Supplementary Table 3. Results of Goal Attainment Scale.

Examples of specific and measurable goals

Motor function

- I want enough energy to perform two hours of activities when I finish my school / university activities (1 hour of homework, 1 hour of leisure activities)
- I want the mean analogous fatigue score (0= no fatigue at all; 10 = the most severe fatigue I can imagine) to reduce from 8 to 6.
- I want to be able to stand up from sitting position from a regular kitchen / office chair without the use of my hands for support
- I want to be stand up from a sitting position on the floor without support of a chair
- I want to be able to walk 60 m without supporting my pelvis with my hand
- I want to be able to abduct my upper arms (with extended elbows) to 130 degrees. Now this is possible to only 90 degrees. This will be illustrative for upper arm function in daily
- I want to be able to lie down from a sitting position without support of my arms, while my head does not fall down suddenly. On my bed
- I want to be able to take my coat (poncho) on and off independently. In order to do so, I need to be able to lift my arms higher
- I want to be able to move myself in the wheelchair for a few cm to get in a more comfortable position. In order to do so, I need more strength in my arms and hip girdle
- I want to be able to open a 1/l PET bottle with my left hand (that is strongest)
- I want to my diplopia to reduce = I want to have single vision in all directions
- I want to be able to change my position in bed
- I want to be able to scratch my back or neck without having to move to one side (to the left); this is an indicator of muscle strength of my right upper arm and trunk muscles
- I want to be able to raise my left arm to the extent that I can put the left hand on my head without support of my right arm and without moving forward with my head. This plays a role in washing and combing my hair
- I want to stand for a short moment to rearrange my clothes and to do a standing transfer. That was possible until the 2nd stoma operation
- I can drink 500 ml and holding the urine in for at least 45 minutes (the length of a class at university) from the moment the urge kicks in
- I can go out for three hours while standing and dancing without having to sit down
- I can ride my bike without support for 4 km (on a flat road without extreme wind)
- I can take a few steps up without support (e.g. on the street) or walk up the full stairs without support of one railing (e.g. in a house or school)
- I can start taking driving lessons and drive in a regular car
- I am able to perform activities (school, sport, leisure activities 3 day parts (morning, afternoon, evening) instead of two day parts, five days in a row (a school or working week)
- I want to able to go to school with public transportation and not have the same fatigued feeling in my legs when I come home
- I want to able to go shopping with my friends for 5 hours (go there by car; 30 min break) and not have the same fatigued feeling in my legs when I come home
- I want to be able to walk faster (to be able to keep up with other of my age) for at least 5 - 10 minutes (the time we walk at school between differences)
- I want to restart playing tennis; this implies that I am able to run to reach a ball. I want to play

with friends

- I want to be able to walk the regular stairs at home (12-13 steps directly) without support of my arms
- I want to be able to perform 2 lunges without support of my hands
- I want to be able to perform 2 squads effectively while keeping my balance
- I want to be able to jump 10 times from a standing position, from my forefeet and get off the floor at least 5 cm
- I want to be able to do step up a higher step (40 cm) without supporting my leg with my hands. E.g. holiday in Spain; walking on rocks; only possible with pressing her legs down
- I want to get up from a kneeling position (one knee on floor) without support of her hands; Example: cleaning the floor after working in the supermarket / closing the laces of her shoes and getting up again.
- Climb up stairs without railing
- Being able to run
- Do outdoor activities with family (being able to walk uphill on uneven trail)
- Being able to bike on a specific road going up home
- Carry heavy objects (for example a box of 20 beers 0,5)
- Able to climb stairs at home consistently with support of one hand
- Improve grip strength to consistently open a bottle lid with her left hand
- Able to move into sitting from supine lying without turning to the side
- 20 Minutes walking distance with aid
- Opening jars, bottles without the use of aids
- Obtain biking on uneven levels
- Feeling secure while walking; able to walk without fear and secure from front door to parked car without walking aid
- Able to ride her bike again: being able to bike in good weather for 15 minutes feeling secure
- More strong in her fingers: able to open up a bottle of soda or a carton of milk without help
- To be able to raise form a chair.
- When climbing stairs: R leading leg when ascending stairs, L lifted up behind currently with effort (dragged)
- To improve walking stability when walking with 1 walking stick in his garden.
- To lift arms above shoulder level
- To be able to work on the computer for more than 3 hours in one session, currently needs rest after 3 hours.
- To be able to roll to the left side independently like he currently does to the right side
- To be able to sit upright at 90 degrees hip flexion for 10-20 seconds.
- Be able to climb stairs easily (13 steps)
- Be able to run 100m without tripping
- Be able to run on the spot
- To complete a 30m body pump class with 6kg weight (total), currently using 3.5kg
- To be able to get up off the floor in one motion, unaided (now object or legs)
- Raising from sitting position at noon without vertical support
- Long walks of at least 2 hours, outdoors, with enough feeling of stability
- I want to go cycling for at least 30 minutes without feeling exhausted
- I want to be able to carry heavy shopping bags without difficulty
- Walking for 2 hours unassisted on sloped

• Independent for social activities, e.g. restaurant, visiting museum<br><br>**Respiratory**<br>• I want to be able to walk 5000 m in my own pace without running out of breath<br>• I want to be able to lay down in my bed at night without use of my inhalator (Foster)<br>• I want to be able to lie down for 30 minutes before I start using my face mask for nocturnal ventilation without my mouth piece<br>• I want to be able to lie flat during the day without shortness of breath for 15 minutes without NIV (for investigations etc.) or during the night with NIV<br>• I want to be able to put on my coat while sitting without being very fatigued and being breathless.<br>• I want to be able to talk long sentences (20 words) without pausing for taking another breath. Currently, I can say approximately 10 -12 words before taking a new deep breath.<br>• I will have stable or improved results on respiratory function tests<br>• I want to be able to blow a balloon (regular size)<br>• Improvement of exercise dyspnea (walking up 2 floors without dyspnea)<br>• To be able to stay up later than 21:30 without use of NIV (increase by 30min)<br>• Achieving stable lung function tests and muscle strength<br><br>**Swallowing**<br>• I can drink or eat any of the following without having to throw up: a small banana milkshake (McDonalds), half a pizza, and 1 apple
Examples of generally defined goals
**Motor function**<br>• Climbing stairs<br>• Walking stability<br>• Muscular strength/function hands<br>• Reduced ptosis<br>• Climbing stairs<br>• Walking stability<br>• Hand strength/function<br>• Reduced ptosis<br>• Improved walking stability<br>• Improved core strength and reduced back pain<br>• Improved mobility<br>• Improvement in climbing and descending stairs<br>• Climbing stairs<br>• Getting up from floor<br>• Longer distance in walking<br>• Carrying heavier loads<br>• Faster and more time handwriting<br>• More endurance<br>• increase walking distance<br>• Improve walking

- Improve muscle strength around knee
- Improve muscle strength of shoulder and arms
- Improve cardiovascular system
- Climbing stairs

Respiratory

- Reduced hypophonia
- Improved speech
- Ability to perform firefighters' breathing apparatus examination
- Reduced exercise intolerance, when respiratory reserve is needed

Swallowing

- Swallowing solid foods

Supplementary table 4. Results of muscle ultrasound.

Mode	Results	Conclusion
Quantitative + semi-quantiative	Significantly increased echo intensity of the m. biceps brachii and m. vastus lateralis on both sides. No atrophy.	*Conclusion:* Abnormal muscle echo consistent with a neuromuscular disorder. The m. biceps brachii and m. vastus lateralis (both sides) seem suitable for possible muscle biopsy.
	Quantitative assessment: markedly increased echo intensity from both deltoid, biceps brachii, vastus lateralis, tibialis anterior, and gastrocnemius medial heads. Mild atrophy of the m. vastus lateralis bilaterally. Semi-quantitative assessment: markedly increased echogenicity in all muscles examined with prominent (eg in the biceps) central muscle a more pronounced increased echogenicity and distorted muscle architecture than peripheral. The m. vastus lateralis is atrophic on both sides and seems most suitable for a biopsy on the distal right (see also video). Alternatively, the biceps brachii muscle could be biopsied bilaterally.	*Conclusion:* Muscle ultrasound in the context of the DYN101 study. Abnormal muscle ultrasound consistent with a neuromuscular disorder. The m. vastus lateralis is atrophic on both sides and seems most suitable for a biopsy on the distal right (see also video). Alternatively, the biceps brachii muscle could be biopsied bilaterally.
	Strongly increased echo intensity of the m.deltoideus bilaterally, m.biceps brachii bilaterally and m.tibialis anterior left. Slightly increased echo intensity of the m.vastus lateralis on the left and m.tibialis anterior on the right. In-norm echo intensity of the m.vastus lateralis right. Atrophy of the m.tibialis anterior bilaterally. Possible atrophy of the m.biceps brachii bilaterally and m.deltoideus bilaterally given the lack of a clear bone echo.	
	Strongly increased echo intensity of the m.deltoideus bilaterally, m.biceps brachii bilaterally and m.vastus lateralis left. Slightly increased echo intensity of the m.vastus lateralis on the right and m.tibialis anterior on both sides. No obvious atrophy of the measured muscles. On visual inspection, the m. vastus lateralis on the right is suitable for a biopsy. On the left, the vastus lateralis is more clearly affected, especially the superficial layer of the muscle. This side is also suitable for biopsy. See also images.	
	Strongly increased echo intensity of the m. biceps brachii bilaterally and m. vatus lateralis bilaterally. NB videos have been made of all muscles; scanned from distal to proximal.	*Conclusion:* Muscle ultrasound in the context of the Dynacure study. All 4 muscles examined have an abnormal ultrasound

		aspect, consistent with a neuromuscular disease. The distal one-third (see also video) of the m. vastus lateralis on the right seems suitable for a muscle biopsy.
	Quantitative assessment: Significantly increased echo intensity from both biceps brachii and vastus lateralis muscles. No atrophy. Semi-quantitative assessment: Muscles were assessed over the entire range to assess suitability for a potential muscle biopsy. All examined muscles have abnormally increased echogenicity and are not evidently atrophic. A scar from an earlier muscle biopsy is still clearly visible in the course of the m. vastus lateral on the right. The vastus lateralis on the left would be a suitable muscle for a possible new biopsy.	*Conclusion:* Muscle ultrasound in the context of the DYN101 study. The muscle ultrasound is abnormal and consistent with a neuromuscular disorder. The m. vastus lateralis on the left, but also the m. biceps brachii on both sides seem suitable for a possible muscle biopsy.
	Quantitative assessment: markedly increased echo intensity of the left deltoid muscle and both biceps brachii, vastus lateralis, and tibialis anterior muscles, and slightly increased echo intensity of the right deltoid muscle. No atrophy.	*Conclusion*: Muscle echo in the context of the DYN101 study. The muscle ultrasound is abnormal and consistent with a neuromuscular disorder. The m. vastus lateralis on the left, but also the examined (visually clearly less affected) arm muscles impress, suitable for taking a muscle biopsy.
semi-quantitative	Semi-quantitative assessment of the biceps brachii and vastus lateralis muscles on both sides. All muscles have abnormally increased echogenicity and are not evidently atrophic.	*Conclusion:* Abnormal muscle ultrasound, consistent with a neuromuscular disorder. The m. biceps brachii and m. vastus lateralis (both sides) seem suitable for possible muscle biopsy.
	Semi-quantitative assessment of the biceps brachii, deltoid and vastus lateralis muscles on both sides. All muscles, but mainly the m. deltoid and m. vastus lateralis on both sides, have abnormally increased echogenicity and are not very atrophic.	*Conclusion:* Abnormal muscle ultrasound, consistent with a neuromuscular disorder. The m. deltoid and m. vastus lateralis seem (both sides) suitable for possible muscle biopsy.
	M. biceps brachii c.m. R 3; M. biceps brachii c.m. L 3;	*Conclusion:* If muscle biopsy

	M. biceps brachii c.l. R 4; M. biceps brachii c.l. L 4; M. vastus lat. R 4; M. vastus lat. L 4. Heckmatt III-IV in all muscles that were examined.	will be performed, left or right biceps brachii muscle medial part should be selected.
	Ultrasound imaging of the both thighs (vastus lateralis) and biceps brachii muscles: Right vastus lateralis: There is completely white muscle with loss of recognisable texture and strong beam attenuation so that the underlying bone is (almost) invisible. Mean greyscale is measured 185. Left vastus lateralis: There is completely white muscle with loss of recognisable texture and strong beam attenuation so that the underlying bone is (almost) invisible. Mean greyscale is measured 175. Right biceps: There is completely white muscle with loss of recognisable texture and strong beam attenuation so that the underlying bone is (almost) invisible. Mean greyscale is measured 125. Left biceps: There is completely white muscle with loss of recognisable texture and strong beam attenuation so that the underlying bone is (almost) invisible. Mean greyscale is measured 135.	*Conclusion:* Findings are in keeping with Heckmatt grade 4 of bilateral vastus lateralis and biceps muscles.
	Ultrasound imaging of the both thighs (vastus lateralis) and biceps brachii muscles. Right vastus lateralis: There is marked increased muscle echogenicity with some loss of muscle architecture and some attenuation of the ultrasound beam to an extent that the underlying bone is less well visible. Heckmatt grade 3. Left vastus lateralis: There is marked increased muscle echogenicity with some loss of muscle architecture and some attenuation of the ultrasound beam to an extent that the underlying bone is less well visible, however slightly better visible as when compared to the right vastus lateralis. Heckmatt grade 3. Right biceps brachii muscle: There is completely white muscle with loss of recognisable texture and strong beam attenuation so that the underlying bone becomes invisible. Heckmatt grade 4. Left biceps brachii muscle: There is completely white muscle with loss of recognisable texture and strong beam attenuation so that the underlying bone becomes invisible. Heckmatt grade 4.	*Conclusion:* Severe muscle atrophy of the biceps brachii and vastus lateralis muscles as described above. The left vastus lateralis muscle is felt most suitable for muscle biopsy
	Ultrasound imaging of the both thighs (vastus lateralis) and biceps brachii muscles: There is severe muscle architectural distortion and increased echogenicity of the vastus lateralis and biceps muscles on both sides. The right vastus lateralis demonstrates completely white muscle with loss of recognisable texture and strong beam attenuation so that the underlying bone is	*Conclusion:* All measured sites would be compatible with Heckmatt grade 4.

	(almost) invisible. Mean histogram grey scale value of 141 measured. The left vastus lateralis demonstrates completely white muscle with loss of recognisable texture and strong beam attenuation so that the underlying bone is (almost) invisible. Mean histogram grey scale value of 141 measured. The right biceps musculature demonstrates completely white muscle with loss of recognisable texture and strong beam attenuation so that the underlying bone is (almost) invisible. Mean histogram grey scale value of 150 measured. The left biceps musculature demonstrates almost completely white muscle with loss of recognisable texture and strong beam attenuation so that the underlying bone is (almost) invisible. Mean histogram grey scale value of 147 measured.	
	Lateral vastus R: Heckmatt grade 4; Lateral vastus L: Heckmatt grade 4; Biceps brachii R: Heckmatt grade 4; Biceps brachii L: Heckmatt grade 4	
	Lateral vastus R: Heckmatt grade 4; Lateral vastus L: Heckmatt grade 4; Biceps brachii R: Heckmatt grade 4; Biceps brachii L: Heckmatt grade 4	
	Lateral vastus R: Heckmatt grade 3; Lateral vastus L: Heckmatt grade 3; Biceps brachii R: Heckmatt grade 4; Biceps brachii L: Heckmatt grade 4	
Qualitative	Both BB and VL, qualitatively, abnormal, fatty infiltration of muscles, left M. biceps brachii post biopsy	
	Abnormal, consistent with CNM, both BB and VL, qualitatively: abnormal, elevated echogenicity, consistent with CNM	
	Both BB and VL, qualitatively, abnormal	
	Both BB and VL, qualitatively: abnormal	

Supplementary table 5. Results of blood tests for muscle and renal function at screening and at baseline visits

Patient	Visit	Date	Creatine (µmol/L)	Creatine Kinase (U/L)	Creatine Kinase-MB (µg/L)	Cystatin C (mg/L)
	Screening	27-Feb-20	42	37	N/A	0.82
	SAD baseline	3-Mar-20	33	42	N/A	0.74
	Unscheduled	10-Dec-21	39	35	0.5	0.83
	MAD baseline	27-Dec-21	38	34	0.7	0.85
	Screening	9-Mar-20	67	36	N/A	1.01
	Scr Unscheduled	23-Jun-20	62	47	N/A	0.99

SAD baseline	7-Jul-20	62	41	N/A	1.1
Unscheduled 1	21-Dec-21	N/A	41	0	N/A
Unscheduled 2	21-Dec-21	60	40	N/A	1.06
MAD baseline	17-Jan-22	58	37	0.3	1.12
Screening	22-Sep-20	42	67	N/A	1.16
SAD baseline	13-Oct-20	N/A	71	2.8	1.05
Unscheduled	11-Jan-22	39	84	1.4	1.13
MAD baseline	31-Jan-22	37	79	1.4	1.16
Screening	29-Jul-20	45	307	N/A	1.15
Scr Unscheduled 1	17-Aug-20	55	128	N/A	1.15
Scr Unscheduled 2	5-Oct-20	42	183	11.8	1.19
SAD baseline	3-Nov-20	41	131	8.2	1.13
Unscheduled	15-Dec-21	46	272	15.9	1.16
MAD baseline	24-Jan-22	43	202	11.3	1.12
Screening	18-Feb-20	26	103	N/A	0.88
Scr Unscheduled 1	25-Aug-20	30	120	N/A	0.89
SAD baseline	31-Aug-20	31	141	N/A	0.89
Unscheduled 1	23-Nov-21	35	102	1.7	0.93
Unscheduled 2	18-Jan-22	33	118	1.8	0.88
MAD baseline	31-Jan-22	33	105	1.3	0.89
Screening	6-Nov-20	18	41	1.3	0.87
SAD baseline	23-Nov-20	N/A	50	1.2	N/A
Unscheduled 2	26-Jan-22	N/A	59	1.1	0.81
MAD baseline	14-Feb-22	18	38	1.3	0.81
Screening	1-Dec-20	71	400	N/A	1.18
Run-in Visit 1	29-Dec-20	65	294	3	1.21
SAD baseline	15-Feb-21	60	533	5.3	1.18
Screening	20-Sep-21	35	236	6.9	0.88
Scr Unscheduled 2	7-Dec-21	36	301	7.5	0.83
SAD baseline	3-Jan-22	36	211	5.4	0.84
Screening	16-Sep-20	60	95	1.8	0.93
Run-in Visit 1	15-Oct-20	N/A	80	1.3	0.87
Run-in Visit 2	14-Jan-21	N/A	59	1	N/A
SAD baseline	8-Mar-21	58	108	2.4	0.88
Screening	19-Jan-21	59	113	1.1	1.02
Run-in Visit 1	1-Mar-21	63	126	1.9	1.01
Run-in Visit 2	31-May-21	63	75	1.1	1.12
SAD baseline	17-Aug-21	52	172	2.5	1.02
Screening	30-Sep-20	34	120	N/A	0.87
Run-in Visit 1	3-Nov-20	33	143	2.8	0.82
Run-in Visit 2	19-Jan-21	34	113	N/A	0.75

SAD baseline	1-Feb-21	25	106	2.2	0.70
Screening	20-Apr-21	N/A	21	0.2	0.89
Run-in Visit 1	18-May-21	N/A	29	0.3	0.94
SAD baseline	28-Jun-21	19	28	N/A	0.96
Screening	18-Feb-20	62	75	N/A	1.01
Scr Unscheduled 2	11-Jan-21	61	92	0.6	1.06
SAD baseline	8-Feb-21	56	79	1.1	1.04
Screening	30-Sep-20	51	133	N/A	0.85
Run-in Visit 1	3-Nov-20	45	158	2.4	0.82
Run-in Visit 2	25-Jan-21	48	63	1.2	0.74
Run-in Visit 3	13-Apr-21	47	50	0	0.74
Run-in Visit 4	5-Jul-21	52	71	0.3	0.82
Run-in Visit 5	25-Oct-21	48	69	1	0.84
SAD baseline	29-Nov-21	53	71	0.3	0.80
Screening	7-Feb-22	38	61	1.4	1.05
Run-in Visit 1	7-Mar-22	38	61	1.4	1.03
Screening	5-Oct-20	22	85	3.4	0.87
Scr Unscheduled 1	21-Oct-20	25	78	3	0.97
Screening	21-Apr-21	49	43	0	0.89
Screening	14-Mar-22	36	31	0.6	0.86
Scr Unscheduled	22-Mar-22	36	29	0	0.96
Screening	17-Feb-22	40	255	6.9	0.86
Screening	7-Jul-21	45	71	1.4	1.11
Run-in Visit 1	13-Aug-21	46	81	2.3	1.13
Run-in Visit 2	19-Nov-21	48	54	1.4	1.13
Screening	1-Sep-21	72	432	5.7	1.01
Screening	23-Feb-21	33	33	1.1	1.02
Run-in Visit 1	23-Mar-21	34	37	N/A	1.08
Screening	24-Jun-21	30	139	6.4	1.09
Scr Unscheduled 1	14-Dec-21	27	100	N/A	1.04
Screening	23-Mar-22	39	41	N/A	0.92
Screening	24-Jan-22	44	65	0.6	0.92
Run-in Visit 1	10-Mar-22	45	50	0.5	0.99
Screening	15-Mar-22	72	28	0.4	1.14

Reference values [LLN-ULN]: Creatinine [62-106] for males and [44-80] for females; Creatine Kinase [20-200] for males and [20-180] for females; Creatine Kinase-MB [0-5]; Cystatin C [0.47-1.09]. Scr: Screening; ULN: Upper Limit of Normal; LLN: Lower Limit of Normal; Red: value above ULN; Orange: value equal to LLN or ULN; Blue: value below LLN

Supplementary Table 6. miRNA expression in skeletal muscle and serum of individuals with DNM2 or MTM1 mutations.

MicroRNA	Muscle					Serum				
	Control	DNM2	p	MTM1	p	Control	DNM2	p	MTM1	p
**miR-133a-3p**	1.132	.8657	0.4196	1.014	>0.999	1.285	1.083	0.8443	1.2	0.9745
**miR-199-5p**	1.831	0.6047	0.1541	0.4897	0.1560	1.033	0.3485	0.0001	0.7111	0.0768
**miR-206-3p**	1.347	2.792	0.0038	2.659	0.0307	1.789	6.0783	0.1473	4.061	0.2007
**miR-486-3p**	1.067	1.563	0.0526	2.077	0.0171	1.184	2.002	0.0272	1.493	0.4221
**miR-1-3p**	1.89	0.2056	0.0013	0.3076	0.2108	1.37	1.409	>0.9999	1.326	>0.9999
**miR-22-3p**	1.256	0.3599	0.0025	0.4306	0.1958	1.031	0.6792	0.0538	0.8982	0.646
**miR-95-3p**	1.045	0.6524	0.0119	0.5453	0.0221	1.108	1.803	0.0908	1.565	0.3716
**miR-193b**	1.059	1.061	0.9895	1.286	0.6381	0.5377	1.582	0.009	2.022	0.3592

All values shown are mean+/- standard deviation. P values (p) are compared to control values for each group.

Supplementary Table 7. Correlation of muscle and serum biomarkers with MFM32 score.

Variable	N	Pearson's Correlation	R^2	P value
DNM2 muscle (ng/uL)	12	0.0896	0.0080	0.7819
DNM2 plasma (pg/mL)	14	0.1703	0.0290	0.5604
MSTN plasma (pg/mL)	14	0.2857	0.0816	0.3221
Muscle miR-206-3p	13	0.4961	0.2461	0.0847
Muscle miR-22-3p	13	0.4741	0.2248	0.1017
Muscle miR-95-3p	13	0.4111	0.1690	0.1629
Muscle miR-1-3p	13	0.6220	0.3868	0.0232
Serum miR-193b-3p	14	-0.2914	0.0849	0.3142
Serum miR-199-5p	14	-0.1647	0.0271	0.5797
Serum miR-486-3p	14	0.341	0.1163	0.2347

Supplementary table 8. Reasons for screen failure.

	Failure to meet enrolment criteria*	Withdrawal by subject	Study terminated by sponsor	Physician decision
**Subjects**	7	2	2	1

*Study enrolment criteria: Inclusion - Male or female aged ≥ 16 years on the date of signing the main Informed Consent Form (ICF); Have a documented mutation in DNM2 or MTM1; Have a symptomatic CNM in the opinion of the investigator, at least mild to moderately affected, i.e. showing clinical symptoms in at least 2 of the relevant 4 domains that will be investigated in this trial (respiratory, muscle strength, muscle function, and dysphagia), and be ambulatory, i.e. being able to walk 10 steps, if needed with support/assisted. If a subject is non-ambulatory but highly functioning in the view of the investigator, he/she may be included following discussion with the sponsor; Have an understanding, ability, and willingness to fully comply with visit frequency, trial procedures and restrictions, including contraceptive requirements; Able to provide written, signed and dated informed consent/assent to participate in the trial. Parental consent (one or both parents) and an assent for subjects < 18 years may be required per local legislation. Exclusion - Clinically significant liver or renal disease; Presence of significant co-morbidities or conditions other than CNM or clinically significant (CS) findings during screening of medical history, physical examination, laboratory testing, vital signs or ECG recording for which, in the opinion of the investigator and the medical monitor, participation would not be in the best interest of the subject (e.g. compromise the safety or well-being) or that could prevent, limit, or confound the protocol-specified assessments (e.g. taking a muscle biopsy); For female subjects of child-bearing potential: pregnant or breastfeeding, or planning to become pregnant during the trial; Current or past abuse of alcohol or recreational/narcotic drugs (with the exception of caffeine and nicotine), which in the investigator's opinion would compromise the subject's safety and/or compliance with the trial procedures; Currently enrolled in any interventional trial or scheduled to participate in such a trial whilst participating in this trial. Subjects are allowed to participate in registry studies; Current or relevant history of physical or psychiatric illness, any medical disorder that may require treatment or make the subject unlikely to fully complete the trial, or any condition that presents undue risk from the IMP or procedures; Intake of any disallowed therapies as noted in Section 5.5 within 12 weeks before the planned first IMP administration; Known or suspected intolerance or hypersensitivity to IMP ingredients or closely-related compounds, or history of a significant allergic reaction to IMP ingredients as determined by the investigator, such as anaphylaxis requiring hospitalization; Legally incapacitated or have limited legal capacity. Lack of mental capacity to fully understand the protocol requirements and complete all study required procedures.

Supplementary Table 9. Plasma ELISA validation parameters.

	**DNM2 ELISA**	**Myostatin ELISA**
Analytical Dynamic Range	31.25-1000 pg/ml	31.25-2000 pg/ml
Functional Dynamic Range	156.25-5000 pg/ml	125-8000 pg/ml
Analytical LLOQ	31.25 pg/ml	31.25 pg/ml
Functional LLOQ	156.25 pg/ml	125 pg/ml
Parallelism	Up to 1:10 dilution	Up to 1:8 dilution
Dilution Linearity	Up to 1:10 dilution**	Up to 1:16 dilution*
Spike recovery	Average: 87.2%<br>Range: 52.1-111.6%	Average: 95.5%<br>Range: 87.6-99.2%
Accuracy and Precision		
Average intra-assay CV, operator 1	7.3%	1.9%
Average inter-day CV, operator 1	11.3%	23.5%
Average intra-assay CV, operator 2	10.0%	2.3%

Average inter-day CV, operator 2	29.5%	20.3%
Average inter-operator CV	11.0%	1.9%

Note: Analytical LLOQ not corrected for sample dilution factor; Functional LLOQ corrected for sample dilution factor.

**For most samples tested*

**For 3/5 samples tested*

Supplementary figures

Supplementary Figure 1. Longitudinal pulmonary function in percentage of predicted value for age of individuals with mutations in DNM2 or MTM1.

(A) % maximum expiratory pressure (%) MEP and

(B) % maximum inspiratory pressure (%MIP) across follow-up visits.

DNM2 individuals – blue dots, MTM1 individuals - red dots, females - small dots, dashed line, males – large dots, solid line.

Supplementary Figure 2. Baseline muscle strength and motor function in individuals with DNM2 or MTM1 mutations.
(A) Motor Function Measure standing and transfers (MFM32 D1, %): **DNM2 M**, 51 ± 20; **DNM2 F**, 60 ± 25; **MTM1 M**, 36 ± 25; **MTM1 F**, 42 ± 23.
(B) Motor Function Measure axial and proximal mobility (MFM32 D2, %): **DNM2 M**, 96 ± 5; **DNM2 F**, 90 ± 12; **MTM1 M**, 76 ± 25; **MTM1 F**, 72 ± 29.
(C) Motor Function Measure distal motor ability (MFM32 D3, %): **DNM2 M**, 95 ± 8; **DNM2 F**, 93 ± 8; **MTM1 M**, 94 ± 6; **MTM1 F**, 94 ± 5.
(D) Total Motor Function Measure (MFM32 total, %): **DNM2 M**, 78 ± 11; **DNM2 F**, 79 ± 16; **MTM1 M**, 64 ± 19; **MTM1 F**, 65 ± 24.
(E) MyoGrip absolute strength (kg): **DNM2 M**, 15 ± 10; **DNM2 F**, 11 ± 6; **MTM1 M**, 14 ± 8; **MTM1 F**, 19 ± 14.
(F) MyoGrip % predicted: **DNM2 M**, 32 ± 20; **DNM2 F**, 25 ± 19; **MTM1 M**, 30 ± 22; **MTM1 F**, 36 ± 20. Data are presented as mean ± standard deviation.

Supplementary Figure 3. Automated image analysis for myofiber side and internal nucleation. (A) Representative images of biopsies frozen well (left), with an acceptable amount of freezing artifact for analysis (middle), and with unacceptable freezing artifact (right).

(B) The automated analysis algorithm steps involved segmenting the myofibers (left), locating the nuclei (middle), and selecting the fibers with internalized nuclei (right).

(C) Zoomed-in region defined by dotted outline in panel B, showing the original image (left) and detected nuclei (right). Scale = 100 µm.

Supplementary Figure 4. miRNA in serum and muscle of individuals with mutations in DNM2 or MTM1. Muscle (A,C,E,G,I) and serum (B,D,F,H,J) values of **miR-486-3p** (A,B), **miR-1-3-p** (C, D), **miR-22-3p** (E,F), **miR-193b-3p** (G,H), and **miR-95-3p** (I,J). Data presented as normalized relative quantity values (NRQ) in muscle, or corrected NRQ (CNRQ) in serum, ± SEM. Significance determined by ordinary one-way ANOVA with Dunnett’s multiple comparisons test.

Supplementary references

1. McPherson AC, McAdam L, Keenan S, Schwellnus H, Biddiss E, DeFinney A, English K: **A feasibility study using solution-focused coaching for health promotion in children and young people with Duchenne muscular dystrophy**. *Dev Neurorehabil* 2018, **21**(2):121-130.
2. Wijntjes J, van Alfen N: **Muscle ultrasound: Present state and future opportunities**. *Muscle Nerve* 2021, **63**(4):455-466.
3. Lawlor MW, Schoser B, Margeta M, Sewry CA, Jones KA, Shieh PB, Kuntz NL, Smith BK, Dowling JJ, Muller-Felber W *et al*: **Effects of gene replacement therapy with resamirigene bilparvovec (AT132) on skeletal muscle pathology in X-linked myotubular myopathy: results from a substudy of the ASPIRO open-label clinical trial**. *EBioMedicine* 2024, **99**:104894.
4. Mack DL, Poulard K, Goddard MA, Latournerie V, Snyder JM, Grange RW, Elverman MR, Denard J, Veron P, Buscara L *et al*: **Systemic AAV8-Mediated Gene Therapy Drives Whole-Body Correction of Myotubular Myopathy in Dogs**. *Mol Ther* 2017, **25**(4):839-854.
5. Lawlor MW, Beggs AH, Buj-Bello A, Childers MK, Dowling JJ, James ES, Meng H, Moore SA, Prasad S, Schoser B *et al*: **Skeletal Muscle Pathology in X-Linked Myotubular Myopathy: Review With Cross-Species Comparisons**. *J Neuropathol Exp Neurol* 2016, **75**(2):102-110.
6. Reumers SFI, Erasmus CE, Bouman K, Pennings M, Schouten M, Kusters B, Duijkers FAM, van der Kooi A, Jaeger B, Verschuuren-Bemelmans CC *et al*: **Clinical, genetic, and histological features of centronuclear myopathy in the Netherlands**. *Clin Genet* 2021, **100**(6):692-702.
7. Biancalana V, Romero NB, Thuestad IJ, Ignatius J, Kataja J, Gardberg M, Héron D, Malfatti E, Oldfors A, Laporte J: **Some DNM2 mutations cause extremely severe congenital myopathy and phenocopy myotubular myopathy**. *Acta Neuropathol Commun* 2018, **6**(1):93.
8. Durieux AC, Vignaud A, Prudhon B, Viou MT, Beuvin M, Vassilopoulos S, Fraysse B, Ferry A, Lainé J, Romero NB *et al*: **A centronuclear myopathy-dynamin 2 mutation impairs skeletal muscle structure and function in mice**. *Hum Mol Genet* 2010, **19**(24):4820-4836.
9. Susman RD, Quijano-Roy S, Yang N, Webster R, Clarke NF, Dowling J, Kennerson M, Nicholson G, Biancalana V, Ilkovski B *et al*: **Expanding the clinical, pathological and MRI phenotype of DNM2-related centronuclear myopathy**. *Neuromuscul Disord* 2010, **20**(4):229-237.

Annex 1

DYN101-C101 Study Muscle Ultrasound Biopsy Guidance

Standard Operating Procedure (SOP)

DYN101-C101 Study Muscle Ultrasound Biopsy Guidance

Standard Operating Procedure (SOP)

List with Abbreviations

ASIS	Anterior superior iliac spine
BB	Biceps brachii
L	Left
MSK	Musculoskeletal
R	Right
ROI	Region of interest
VL	Vastus lateralis

Introduction

Muscle ultrasound imaging will be performed at screening to determine which muscle should be biopsied. This imaging should not be older than 6 months at baseline, otherwise it should be repeated. See trial schedules for further details on timing.

Muscle biopsies should be obtained from the vastus lateralis according to the site's standard practice (open or needle biopsy) if sufficient muscle mass is identified after ultrasound imaging. In the event inadequate muscle tissue is identified in the vastus lateralis after ultrasound imaging, a biopsy should be obtained from the biceps brachii (open or needle biopsy).

A. Preparation prior to start of the study

Ultrasound equipment and presets

Linear probe with ≈ 10 MHz center frequency

Fixed MSK presets for both muscles (vastus lateralis/VL and biceps brachii/BB), fixed gain and focus settings

- Preset VL: 6 cm depth
- Preset BB: 4 cm depth

Measure control subject healthy VL and healthy BB, adjust gain to create a mean greyscale level for VL of 50-90 (on a scale from 0-255) and for BB of 30-70 (0-255); see below.

Save both setting as a dedicated preset. Keep all parameters constant during scanning and image capturing, do NOT adjust gain, time-gain controls, depth, focus or other image optimization during scanning.

During scanning, make sure the probe is perpendicular to the underlying bone at an exact 90 degree scan angle. Check this by tilting the probe slightly to find the angle with the brightest bone reflection.

Measurement protocol for VL:

Patient supine, knee extended (or in case of flexion contracture: put small pillow below knee and let leg rest in slightly abducted position)

**Vastus lateralis***	2/3 of the lateral line spina iliaca - upper edge patella	Supine position	Femur to superficial fascia(includes vastus intermedius)

Transducer in transverse position

2/3 of the way along a line from the ASIS to upper lateral margin of patella, femur in center of image with optimal bone echo

The muscle needs to be equally thick at either side of the image

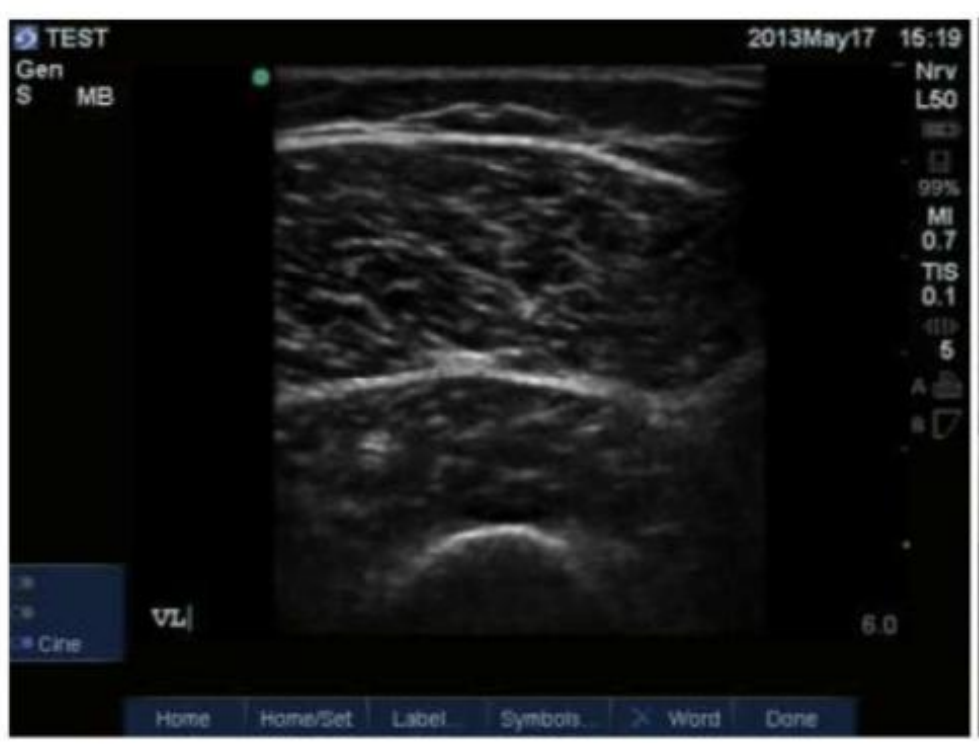

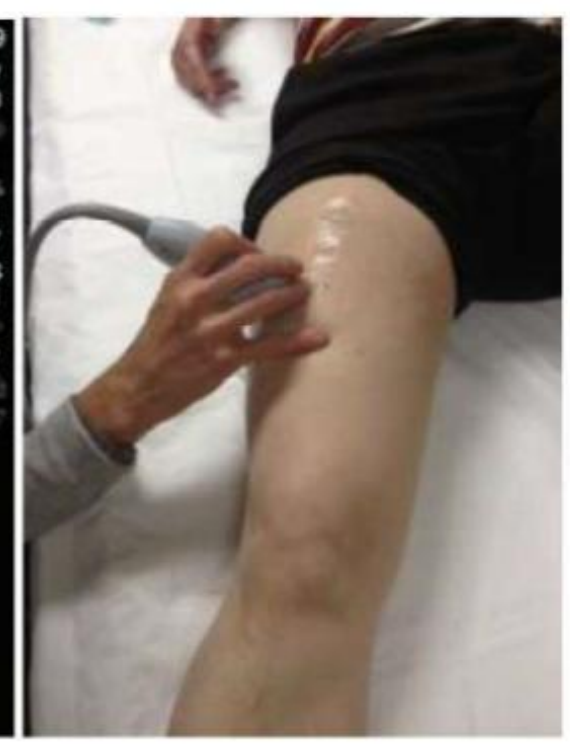

Ideally, the VL image should look like this:

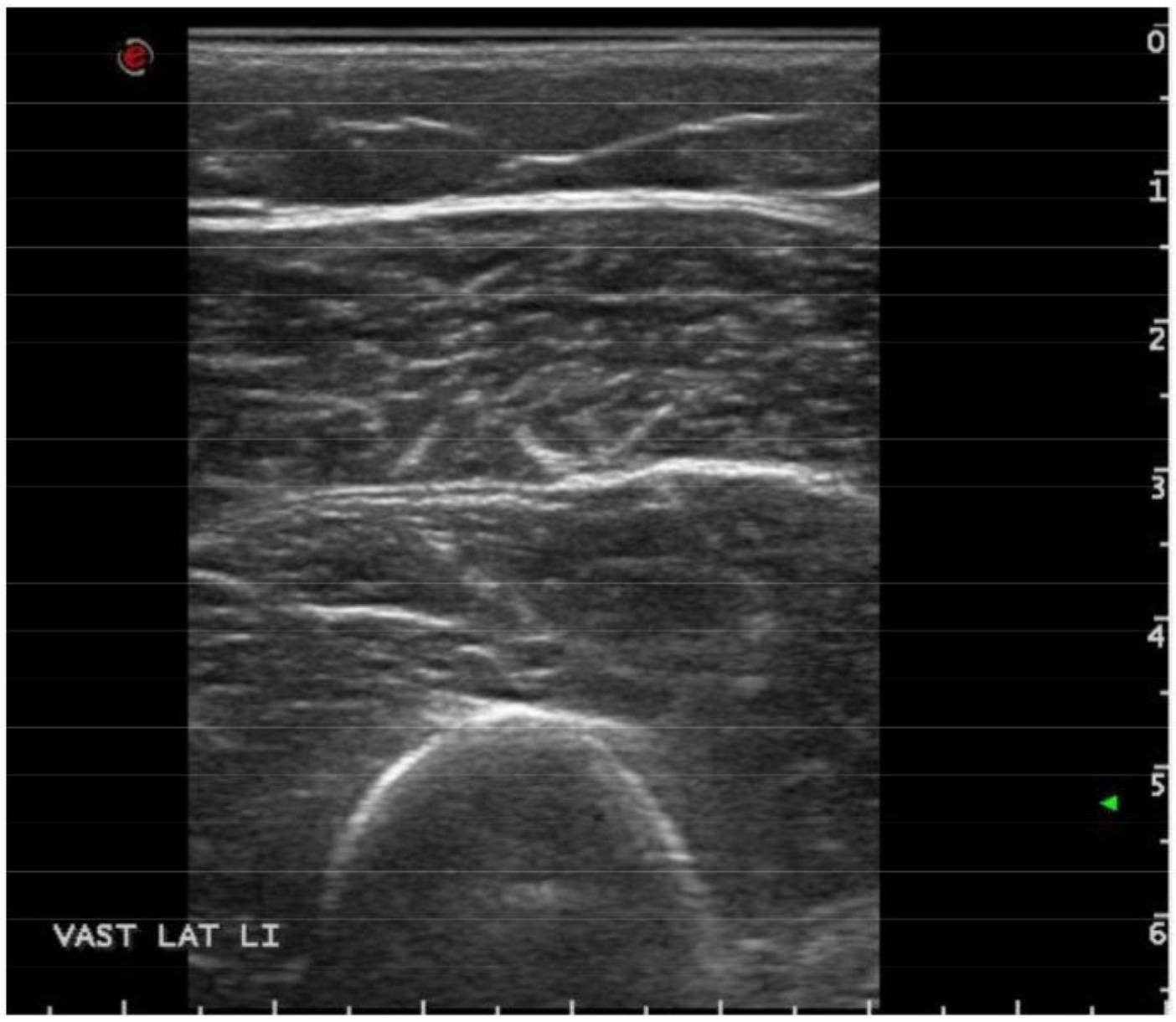

Measurement protocol for BB:

Patient supine, forearm supinated (or in case of elbow flexion contracture: patient in right lateral decubitus position with forearm supported by small pillow)

Muscle	Location	Positioning	Thickness measurement
**Biceps brachii**	2/3 of the line acromion - antecubial fossa	Supine position	Humerus to superficial fascia(includes brachialis)

Measurement location: 2/3 of the distance from acromion to antecubital crease

In the picture the humerus is located in the middle

Find the neurovascular bundle and keep that just on the edge of the picture

Make sure you don't press down, so muscle is still curved (need heaped up gel)

Ensure there is no black shadowing

Make sure bone echo is bright, and that muscle is at its maximal whiteness as well

Measure biceps and brachialis for thickness

Place the ROI rectangle in the upper part of the biceps muscle

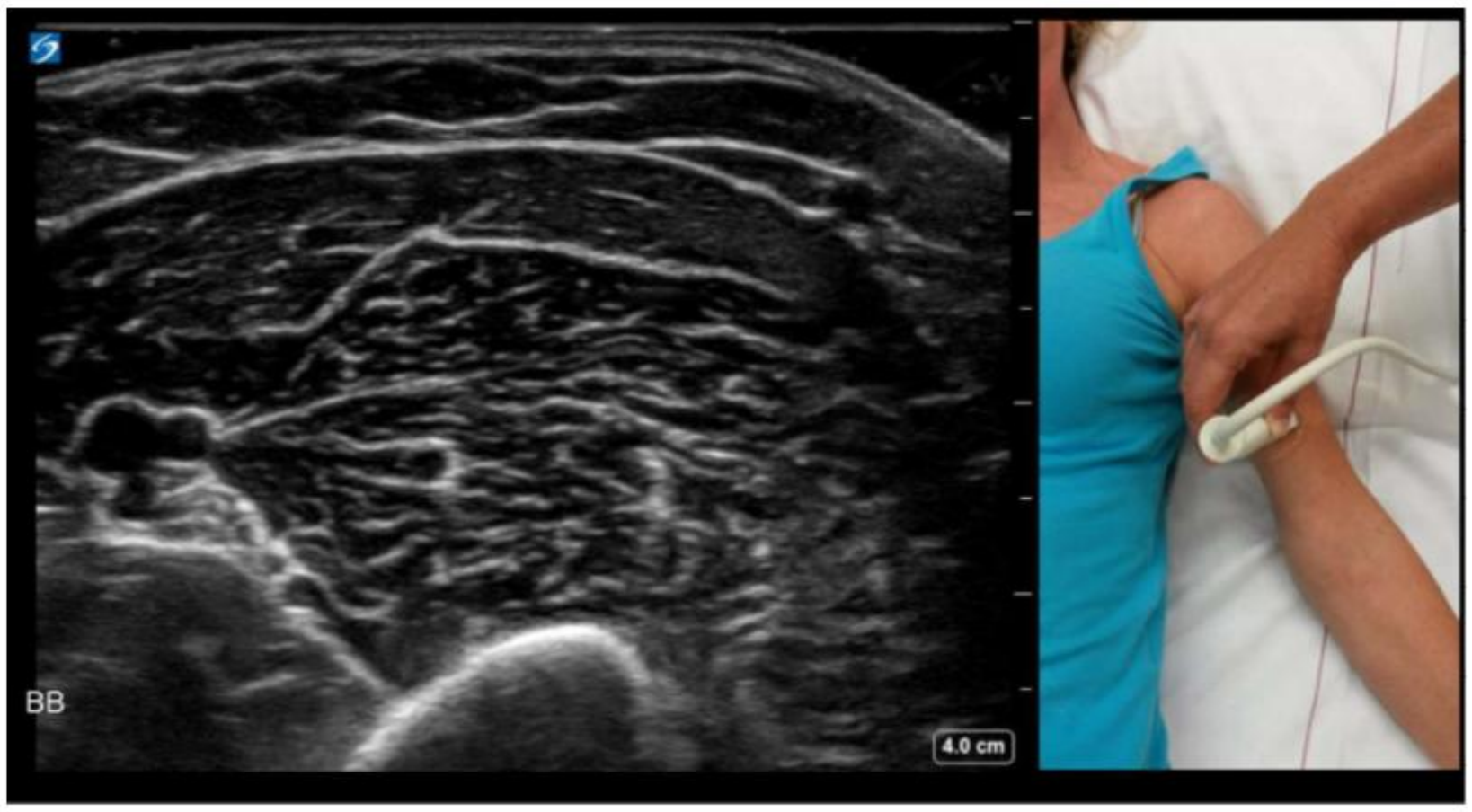

Ideally, the BB image should look like this:

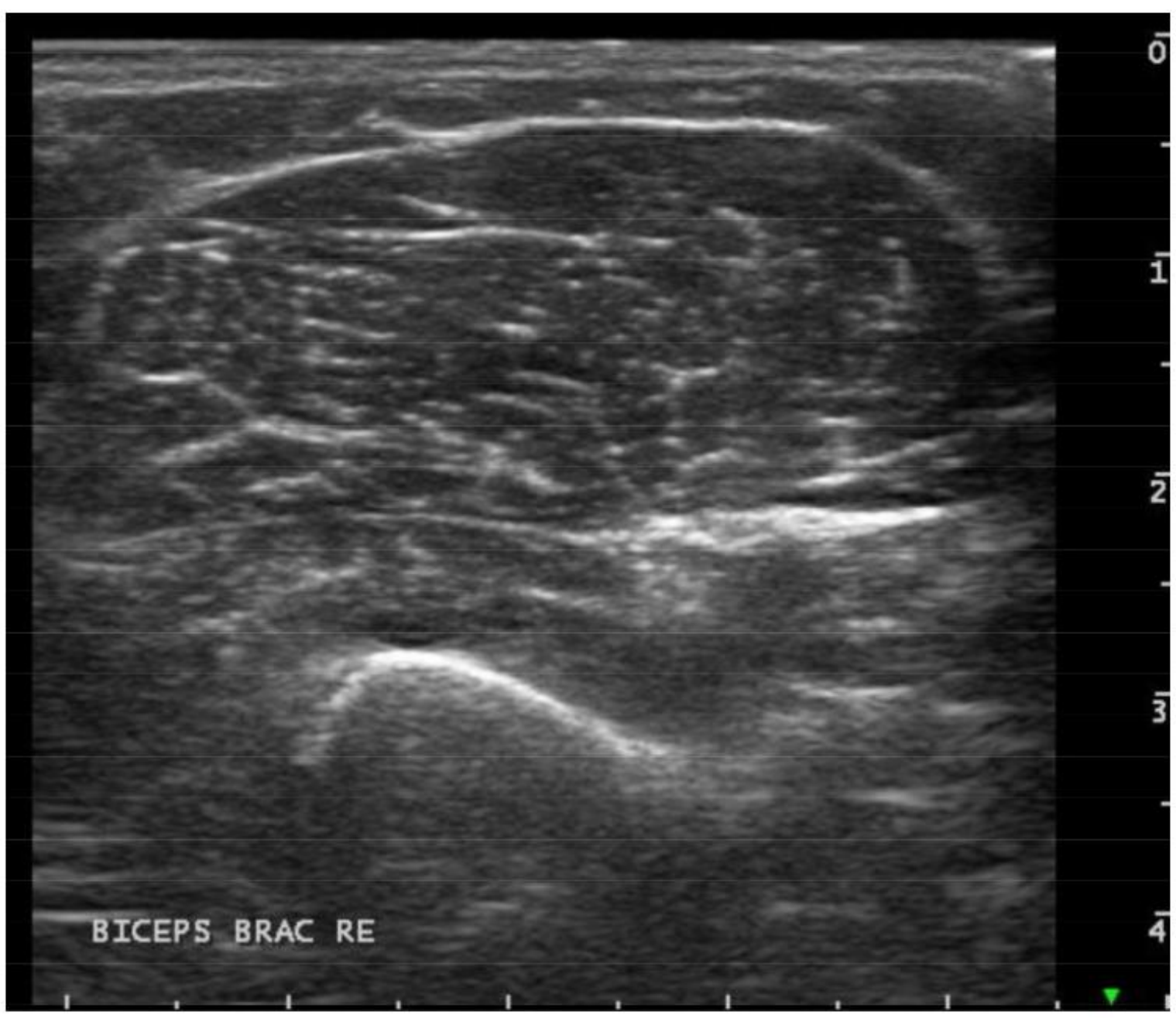

To find the mean greyscale levels of your sample images:

Download ImageJ free software from NIH: https://imagej.nih.gov/ij/.

Open program, select image file using "Open…" command in file menu.

Select a region of interest using the "Rectangle" tool icon (below the File menu) to place the ROI rectangle in the image, include BB and brachialis muscle but avoid subcutis, bone and the lateral 1 cm of the images.

Select the "Histogram" option from the "Analyze" menu. Note the mean value from the pop-up panel; this should ideally be in the 50-90 range for the VL and 30-70 range for the BB in a healthy subject.

See sample screenshots below (note: mean greylevels are a bit lower than preferred in these sample images).

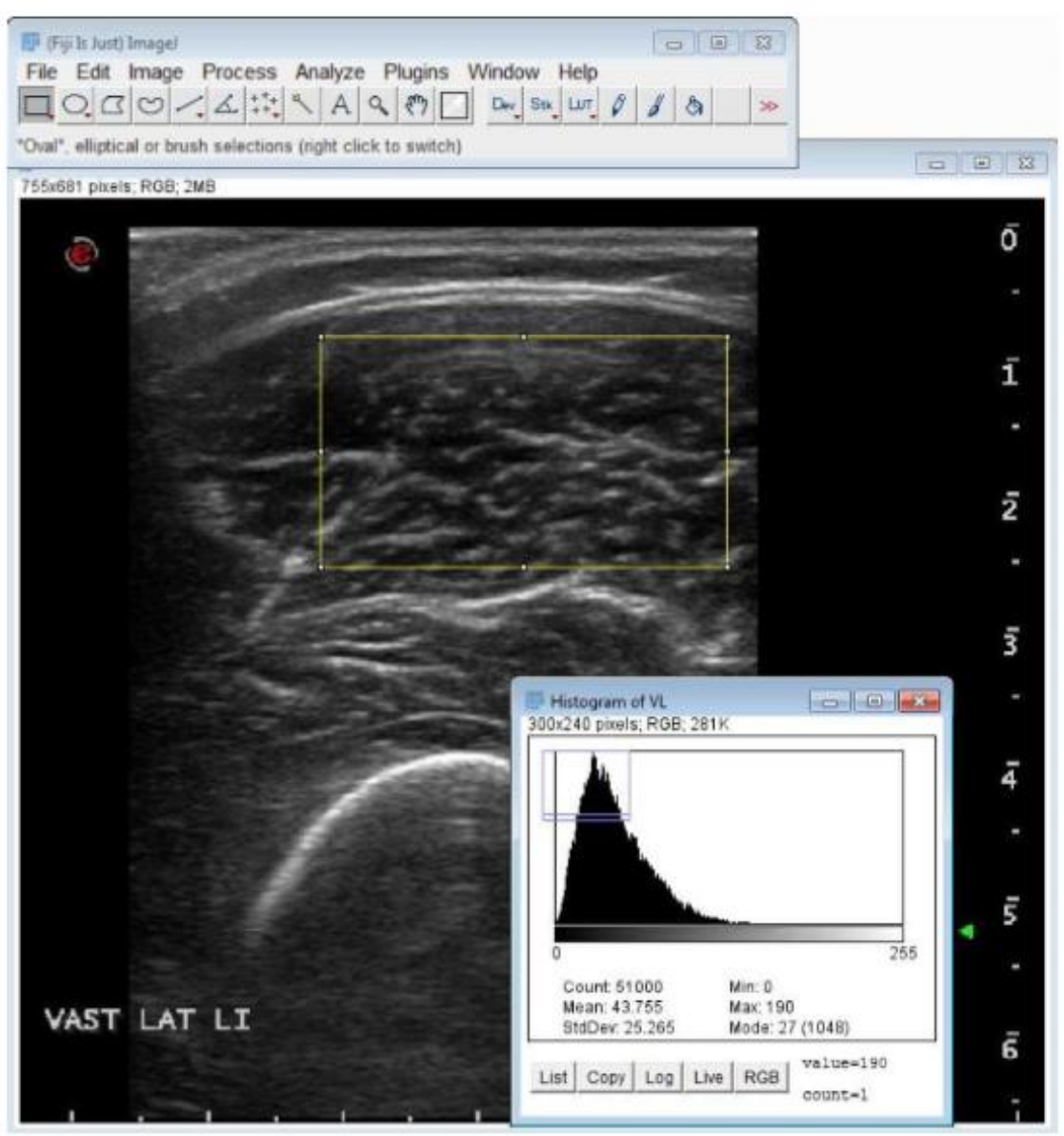

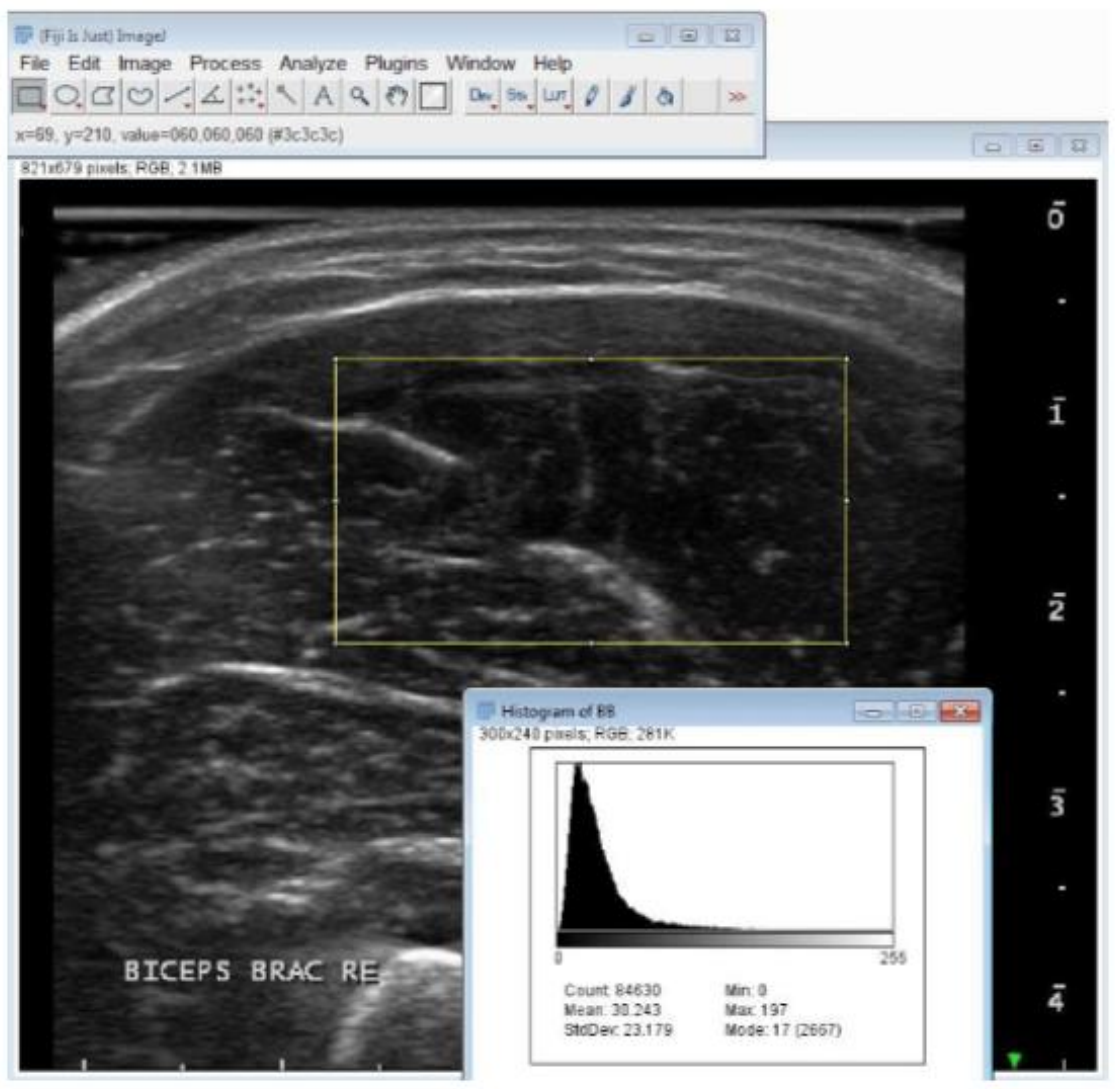

Save image + ROI + histogram with a screencapture image and send to study ultrasound consultant. If you are having difficulty obtaining the mean greylevels in this manner, consult your study contact for feedback from the study ultrasound consultant.

B. Muscle selection for biopsy with ultrasound

Primary muscle of choice: vastus lateralis (VL)

Scan both left and right VL, using fixed presets and protocols as described above; do NOT adjust any image parameters during scanning.

Annotate images: VL for vastus lateralis; add “R” for right side; add “L” for left side. Put the annotation in the lower left corner of the image, not in the muscle itself.

Visually evaluate the images using Heckmatt grading scale (see below).

If the vastus lateralis is found unsuitable (mainly: too affected, see below): select biceps brachii (BB)

Scan both left and right BB, using fixed presets and protocols as described above; do NOT adjust any image parameters during scanning.

Annotate images: BB for biceps brachii; add “R” for right side; add “L” for left side. Put annotation in lower left corner of image, not in the muscle itself.

Visually evaluate image using Heckmatt grading scale (see below).

How to grade muscle quality for biopsy using the Heckmatt grading scale:

Heckmatt grading is done by judging 3 aspects of the muscle in the image:

- Is there increased echogenicity, i.e. an increased greylevel of the muscle tissue of interest? As a rough reference, normal muscle should be of similar echogenicity as the subcutaneous fat layer.
- Is the muscle texture changed, i.e. is the muscle of interest still recognizable as muscle tissue on a transverse image, with typical fascial structures?
- Is there attenuation of the ultrasound beam by an increase in the amount of reflections in the superficial layers of the muscle of interest, i.e. can you still see through the muscle or is the underlying bone echo diminished or absent?

Of note: although unlikely, Heckmatt grading may be affected by other pathology in the muscle, such as traumatic ecchymoses, hematomas or ruptures. If this is suspected clinically, choose the next preferred muscle (first: left and right VL, if both unsuitable: left and right BB).

Grade muscles as follows:

Heckmatt grade 1: normal muscle appearance that is just as black or grey as the overlying subcutis, clearly visible muscle texture (or architecture), minimal attenuation of the ultrasound beam with clear underlying bone reflection

Heckmatt grade 2: slight overall increase in muscle echogenicity (i.e. whiteness), texture still recognizable as muscle, no attenuation of underlying structures

Heckmatt grade 3: further increase in overall echogenicity with some loss of muscle architecture and some attenuation of the ultrasound beam so that the underlying bone is less well visible

Heckmatt grade 4: completely white muscle with loss of recognizable texture and strong beam attenuation so that the underlying bone is (almost) invisible

Here are examples of Heckmatt grade 1 – 4 muscle images for the right VL muscle:

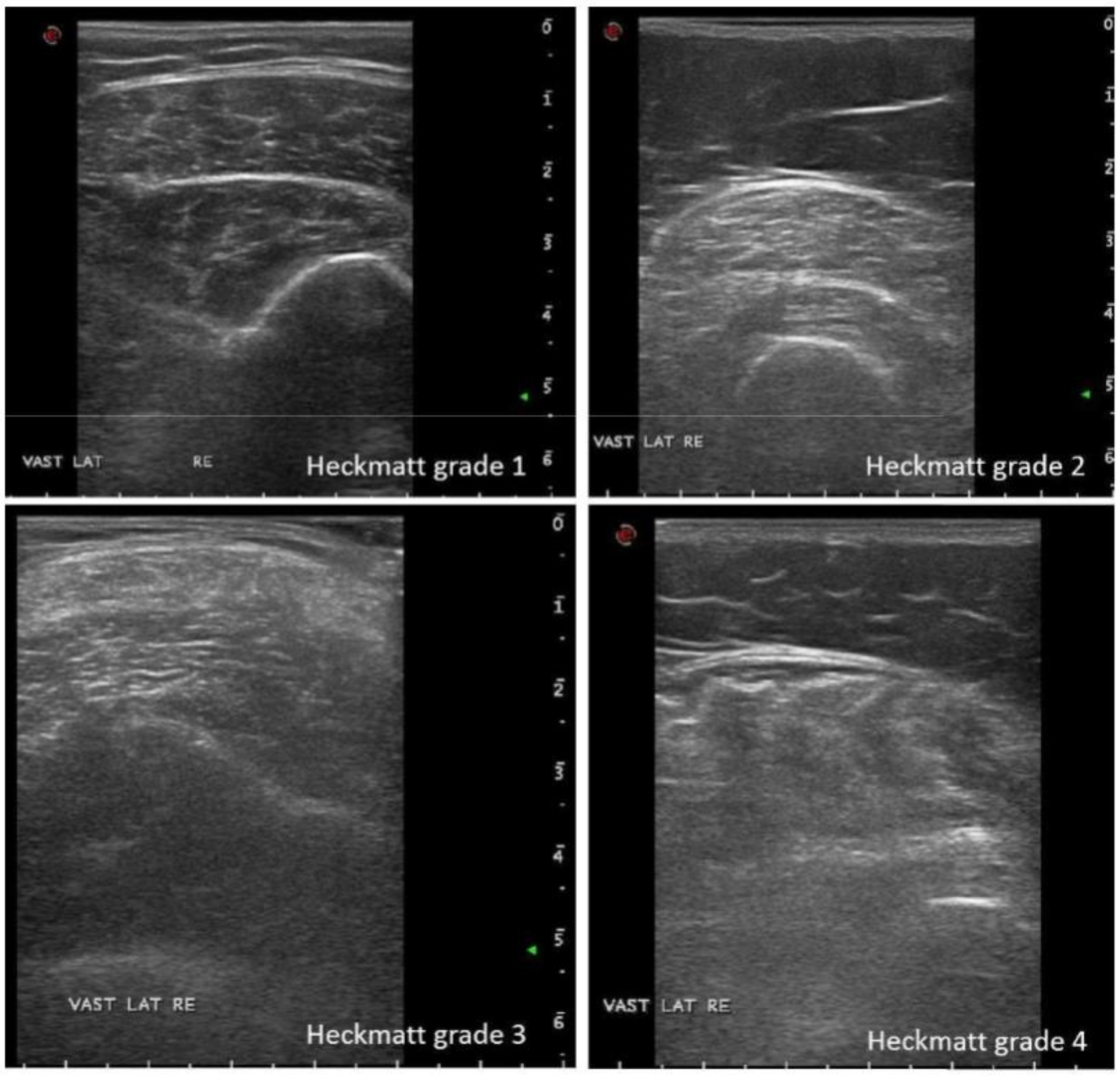

Here are examples of Heckmatt grade 1 – 4 muscle images for the right BB muscle:

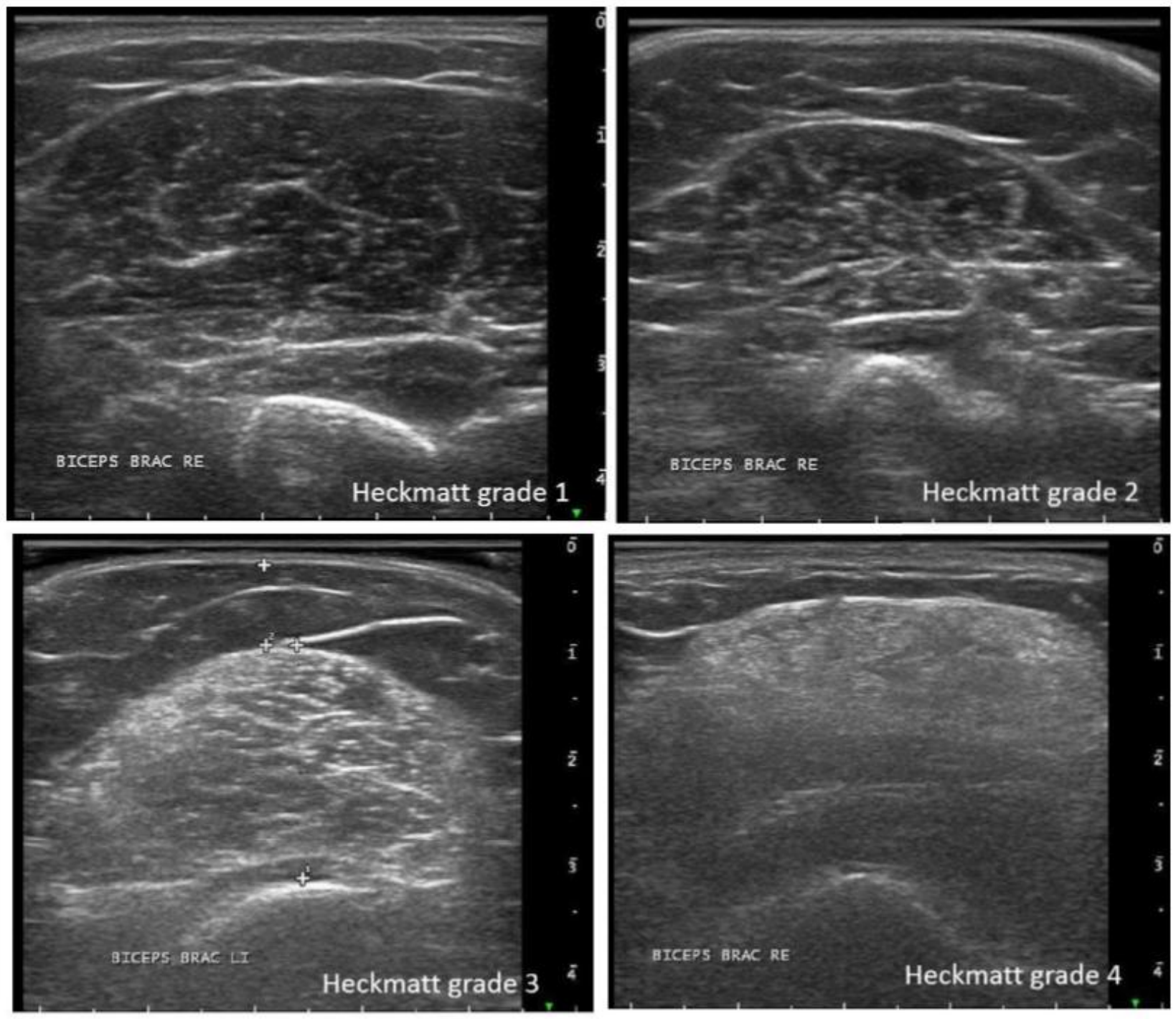

How to select the muscle to be biopsied

For biopsy, only Heckmatt grade 1, 2 or 3 muscles are acceptable; Heckmatt grade 4 muscles are too degenerated and a biopsy will only show fat and/or fibrosis. Preferably, select a Heckmatt grade 2 muscle (or part of the muscle) for biopsy

If right and/or left VL show Heckmatt grading ≤ 3, select VL. Heckmatt grade 2 muscle has the preference over grade 1 and 3 muscles. In the event the ultrasound shows same grading and findings bilaterally, use clinical judgement to finally select L or R VL.

If right and left VL both show Heckmatt grading 4, assess left and right BB. If both sides have Heckmatt grading ≤ 3, preferably select side with Heckmatt grade 2 muscle. In the event the ultrasound shows same grading and findings bilaterally, use clinical judgement to finally select L or R BB.

In the event both left and right VL and left and right BB show Heckmatt grade 4 findings, the preferred biopsy location is the BB. Final selection of the muscle to be biopsied should however be based upon clinical judgement.

Note: in centronuclear myopathies muscles can be focally affected, see sample image of the BB below. Preferentially select a region that is Heckmatt grade 2 for optimal histology (i.e. the left half of this muscle).

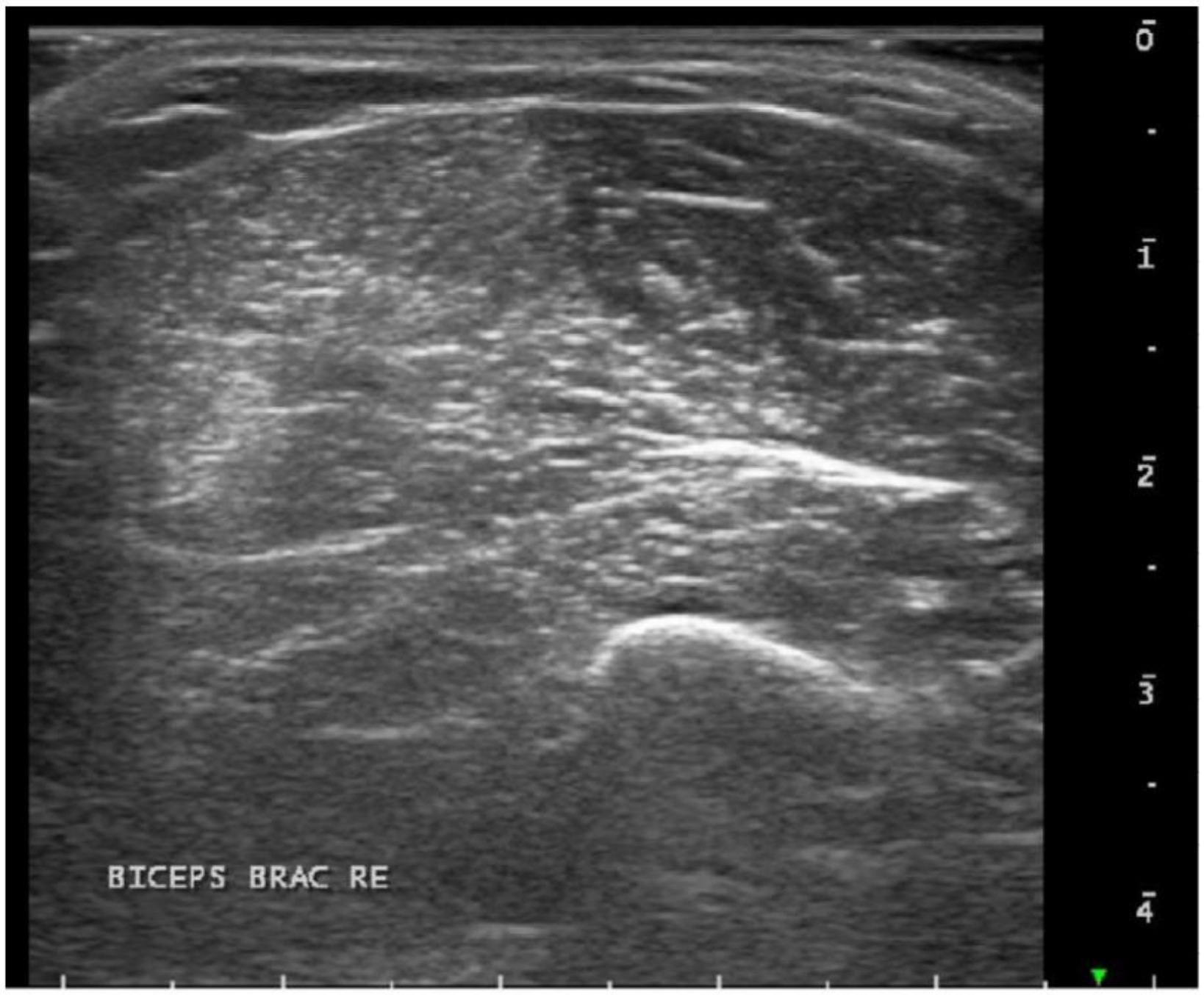

Example: focally affected BB in centronuclear myopathy.

C. Image capturing and storage

After selecting the optimal site for muscle biopsy, capture 3 separate images from the muscle using the prescribed preset and measurement protocol. Take the probe off and put it back on the skin between images, and each time adjust the probe angle anew for the optimal 90 degree bone reflection.

Save images in a .dicom or. Jpg format and send to the Study coordinators.

Addendum: measurement pitfalls

Watch out for these pitfalls:

Angulation of the probe will lead to less than 90 degree scanning angles and a substantially darker image

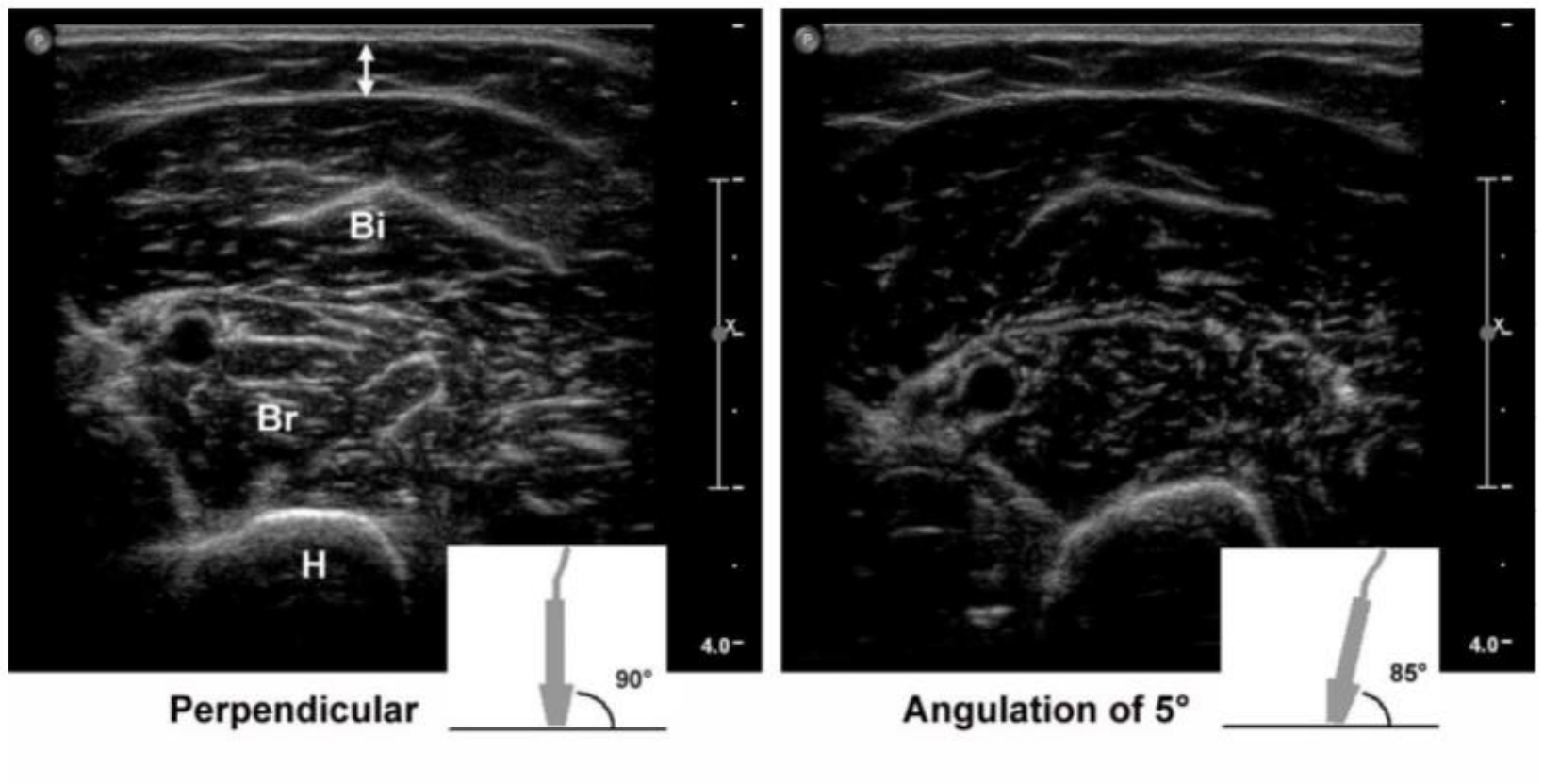

Do not compress the skin (decreases thickness and makes the image whiter), and make sure the patient is fully relaxed (contraction will increase thickness and makes the image darker)

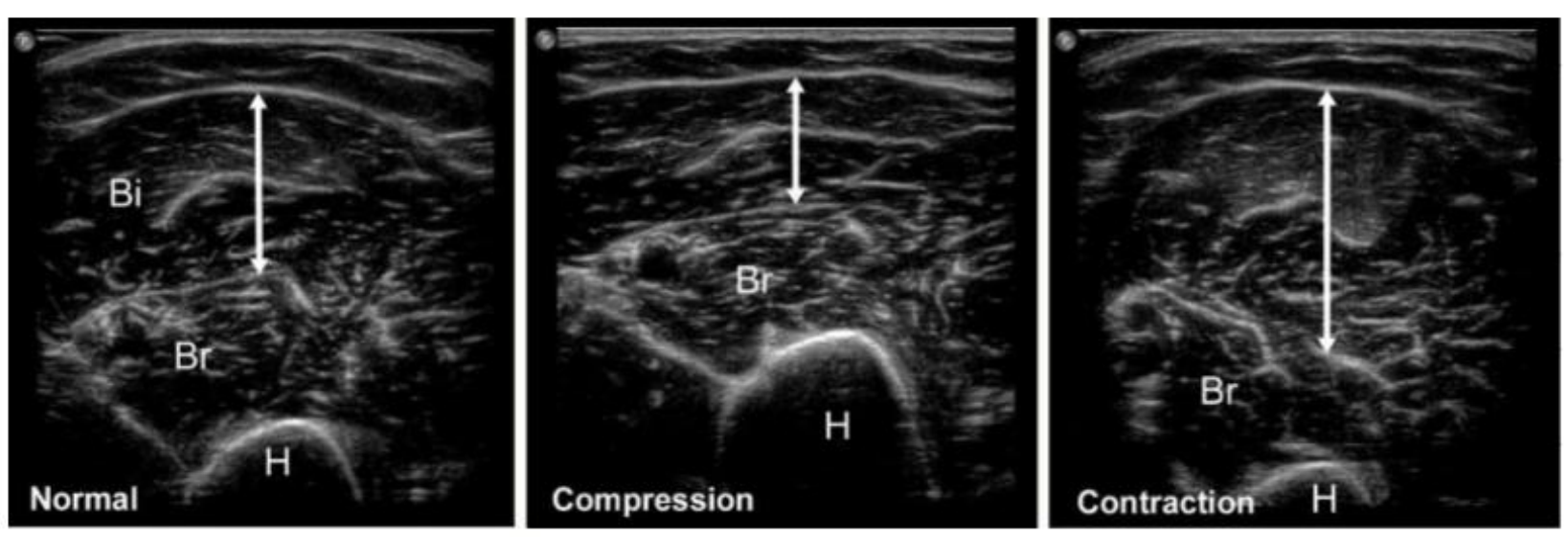

Supplement: Supplementary file 1 [file sj-pdf-1-jnd-10.1177_22143602261471202.pdf]
